# Chemotherapy-induced high expression of IL23A enhances efficacy of anti-PD-1 therapy in TNBC by co-activating the PI3K-AKT signaling pathway of CTLs

**DOI:** 10.1038/s41598-024-65129-7

**Published:** 2024-06-20

**Authors:** Fan Pan, Jiajing Liu, Ying Chen, Binghan Zhu, Weiwei Chen, Yuchen Yang, Chunyan Zhu, Hua Zhao, Xiaobei Liu, Yichen Xu, Xiaofan Xu, Liqun Huo, Li Xie, Rui Wang, Jun Gu, Guichun Huang

**Affiliations:** 1 Department of Oncology, Nanjing Jinling Hospital, Affiliated Hospital of Medical School, Nanjing University, Eastern Zhongshan Road 305#, Nanjing, 210002 China; 2 Department of Breast and Thyroid Surgery, The First Affiliated Hospital of Ningbo University, Liuting Road 59#, Ningbo, 315010 China; 3 Medical School of Nanjing University, Nanjing University, Hankou Road 22#, Nanjing, 210093 China; 4 Department of Respiratory Medicine, Nanjing Drum Tower Hospital, Clinical College of Nanjing University Medical School, Zhongshan Road 321#, Nanjing, 210008 China; 5 Department of General Surgery, Jinling Hospital, Affiliated Hospital of Medical School, Nanjing University, Eastern Zhongshan Road 305#, Nanjing, 210002 China; 6 Comprehensive Cancer Center of Nanjing Drum Tower Hospital, Affiliated Hospital of Medical School, Nanjing University, Zhongshan Road 321#, Nanjing, 210008 China

**Keywords:** Triple-negative breast cancer, Immunotherapy, Immune checkpoints inhibitor, Chemotherapy, IL-23, Cancer, Immunology

## Abstract

Treatment of advanced triple-negative breast cancer (TNBC) is a great challenge in clinical practice. The immune checkpoints are a category of immunosuppressive molecules that cancer could hijack and impede anti-tumor immunity. Targeting immune checkpoints, such as anti-programmed cell death 1 (PD-1) therapy, is a promising therapeutic strategy in TNBC. The efficacy and safety of PD-1 monoclonal antibody (mAb) with chemotherapy have been validated in TNBC patients. However, the precise mechanisms underlying the synergistic effect of chemotherapy and anti-PD-1 therapy have not been elucidated, causing the TNBC patients that might benefit from this combination regimen not to be well selected. In the present work, we found that IL-23, an immunological cytokine, is significantly upregulated after chemotherapy in TNBC cells and plays a vital role in enhancing the anti-tumor immune response of cytotoxic T cells (CTLs), especially in combination with PD-1 mAb. In addition, the combination of IL-23 and PD-1 mAb could synergistically inhibit the expression of Phosphoinositide-3-Kinase Regulatory Subunit 1 (*PIK3R1*), which is a regulatory subunit of PI3K and inhibit p110 activity, and promote phosphorylation of AKT in TNBC-specific CTLs. Our findings might provide a molecular marker that could be used to predict the effects of combination chemotherapy therapy and PD-1 mAb in TNBC.

## Introduction

Breast cancer is the most frequently diagnosed cancer and the fifth leading cause of cancer-related death worldwide^1^. Triple-negative breast cancer (TNBC) is referred to as a subtype of breast cancer that lacks estrogen receptor (ER), progesterone receptor (PR) expression, and human epidermal growth factor receptor 2 (HER-2) expression/amplification. It has an aggressive clinical behavior with poor prognosis^2,3^. Treatment of advanced TNBC is still a great challenge in clinical practice. Cytotoxic chemotherapy is the primary treatment for TNBC patients^4^. However, some TNBC patients still cannot fully benefit from chemotherapy^5,6^.

Immune escape is described as one of the hallmarks of cancer^7^. The B7/CD28 family, which includes co-stimulators and co-inhibitors of immune responses, is closely associated with the function of tumor-specific T-cell immunity in the tumor microenvironment^8^. Among these molecules, the cytotoxic T-lymphocyte antigen 4 (CTLA-4) and programmed cell death 1 (PD-1)/programmed death ligand-1 (PD-L1) are known as immune checkpoints that are essential for maintaining immune tolerance^9^. Antagonists against these signals provide new strategies for enhancing anti-tumor immunity. Interestingly, the immune index, indicated by tumor-infiltrating lymphocyte (TIL) counts or expression of immune-related genes, was significantly higher in TNBC than other subtypes of breast cancer and closely associated with the prognosis of TNBC patients, suggesting the potential efficacy of immune therapy on TNBC patients^10–12^.

The efficacy and safety of the PD-1 monoclonal antibody (mAb) in metastatic TNBC have been demonstrated^13–15^. Notably, combined treatment with pembrolizumab and chemotherapy was shown to enhance the pathologic complete response (pCR) and progression-free survival (PFS) of TNBC patients, suggesting that PD-1 mAb and chemotherapy may have a synergistic anti-tumor efficiency on TNBC patients^16–18^. Previous studies have partially explained the mechanisms by which chemotherapy enhances anti-tumor immunotherapy^19–22^. However, the underlying mechanisms of synergistic anti-tumor effect between chemotherapy and PD-1 mAb have not been elucidated.

The present study found that IL-23 plays a vital role in mediating the anti-tumor immune response induced by chemotherapy in TNBC. We demonstrated that IL-23 synergies with PD-1 mAb in anti-tumor immunity. Mechanically, IL-23 and PD-1 mAb both decrease the expression of Phosphoinositide-3-Kinase Regulatory Subunit 1 (*PIK3R1*), which is a regulatory subunit of PI3K and inhibit p110 activity, thereby promoting the subsequent activation of AKT pathways in tumor-specific cytotoxic T lymphocytes (CTLs).

## Results

### IL23A was significantly upregulated in TNBC cells after chemotherapy

**Figure 1:**
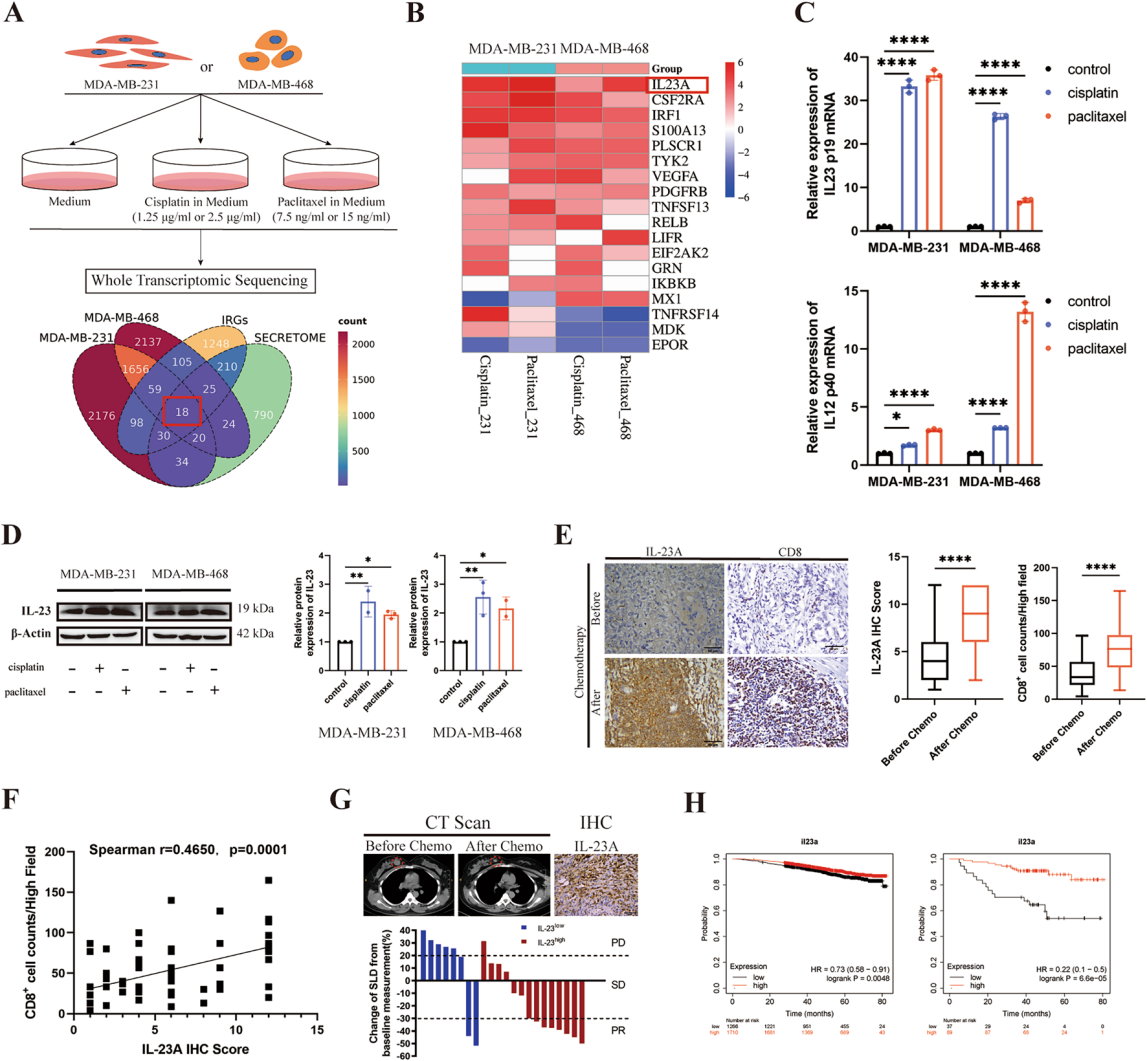
IL23A was significantly upregulated by chemotherapy and associated with a favorable prognosis in TNBC patients. (**A**) The whole transcriptomic sequencing of TNBC cells treated with chemotherapeutic drugs. Upper panel, the experimental design. Lower panel, overlapping immune- and secretome-related genes, and significantly affected genes in TNBC cells treated with chemotherapeutic drugs (log2|Fc|≥ 2, P < 0.05). (**B**) Heapmap of 18 genes identified in Fig. 1A, ranked by normalized log2Fc. IL23A was identified as the most upregulated gene in response to chemotherapeutic drugs. (**C**) mRNA levels of two subunits of IL-23 (IL23A and IL12B) in TNBC cells treated with chemotherapeutic drugs, detected by qRT-PCR. (**D**) Protein expressions and quantifications of IL-23 in TNBC cells treated with chemotherapeutic drugs, detected by western blot. The PVDF membranes were cropped at 35 kDa before antibody incubation. Original blots are presented in Supplementary Fig. S6. (**E**) Left panel, representative immunohistochemistry (IHC) staining of IL-23A and CD8 on resected tumor tissues from TNBC patients who did (n = 27) or did not (n = 37) receive neoadjuvant chemotherapy. Scale bars 50 μm. Middle and right panel, quantitative analysis. (**F**) Spearman correlation analysis revealing a correlation between IL-23A staining score and CD8 + T cell counts in resected tissues from TNBC patients (n = 64, r = 0.465, p = 0.0001). (**G**) Upper panel, representative CT scans before and after neoadjuvant chemotherapy from one of the patients whose resected tumor tissues exhibited high scores of IL-23A staining, as well as the representative IHC staining. Scale bars, 50 μm. Lower panel, the slope chart shows the overall response rate (ORR) of TNBC patients who received neoadjuvant chemotherapy (n = 22). Blue bars represent patients from the IL-23A^low^ group with an IL-23A IHC score of 0–6 (n = 8); Red bars represent patients from the IL-23A^high^ group with an IL-23A IHC score of 7–12 (n = 14). (H) The correlation between IL23A mRNA levels and overall survival of patients with breast cancer (HR = 0.73, P = 0.0048, left panel) or Triple-negative breast cancer (HR = 0.22, P < 0.0001, right panel). Data from the Kaplan–Meier plotter database (https://www.kmplot.com/). Error bars represent standard deviation (SD), *P < 0.05; **P < 0.01; ****P < 0.0001.

To investigate the effects of chemotherapy on TNBC cell lines, we first figured out the dose–response curve for cisplatin and paclitaxel of MDA-MB-231 and MDA-MB-468 cells. Next, we identified the appropriate low doses (1.25 μg/ml of cisplatin and 7.5 ng/ml of paclitaxel) and conventional doses (2.5 μg/ml of cisplatin and 15 ng/ml of paclitaxel) of these two drugs, which resembled clinical practice (Supplementary Fig. S1). Afterward, we conducted the whole transcriptomic sequencing on MDA-MB-231 and MDA-MB-468 cell lines following treatment with low- or conventional- doses of cisplatin or paclitaxel (Fig. 1A). We compared the differentially expressed genes (DEGs) in TNBC cells following treatment with chemotherapeutic drugs (log_2_|Fc|≥ 2, p < 0.05) with immune-related genes (IRGs) obtained from the ImmPort database and secretory molecule-encoding genes^23^. In total, we found eighteen immune- and secretome-related genes, expression of which were significantly changed after chemotherapy (Fig. 1A). Among these genes, IL23A exhibited a distinctive upregulation (Fig. 1B). Further validation using quantitative real-time PCR and Western blot demonstrated that the expression of one subunit of IL-23, p19 (coded by *IL23A*), was significantly upregulated following treatment with cisplatin or paclitaxel in both MDA-MB-231 and MDA-MB-468 cell lines (Fig. 1C,D). In comparison, the expression of the other subunit of IL-23, p40 (coded by *IL12B*), was just minorly upregulated (Fig. 1C).

### Increased expression of IL23A was clinically associated with better outcomes for TNBC patients

To further study the relationship between the expression of IL23A and chemotherapy in TNBC samples, we collected resected tumor tissues from 64 TNBC patients, 27 of whom had previously received neoadjuvant chemotherapy. Our findings revealed that tumor tissues from patients undergoing neoadjuvant chemotherapy displayed higher IL-23A immunohistochemical (IHC) staining scores than those without neoadjuvant chemotherapy. Then, we assessed the infiltration of CD8^+^ T lymphocytes in these TNBC tissues and found that tumor tissues from patients who had received neoadjuvant chemotherapy exhibited a higher level of infiltration of CD8^+^ T lymphocytes (Fig. 1E). Spearman correlation analysis demonstrated a positive correlation between the infiltration of CD8^+^ T lymphocytes and the expression of IL-23A in TNBC tumor tissues (Spearman r = 0.465, p = 0.0001) (Fig. 1F). To explore the relationship between IL23A and chemotherapy efficacy, we divided patients who had received neoadjuvant chemotherapy into two groups based on IL-23A expression levels. Our findings revealed that patients in the IL-23A^high^ group showed a better overall response rate (ORR), indicating a better response to chemotherapy (Fig. 1G, Table 1). Statistics from the Kaplan–Meier plotter database (https://www.kmplot.com) revealed that higher expression of IL23A was correlated with better overall survival in patients with breast cancer (HR = 0.73, P = 0.0048) (Fig. 1H, left panel). The tendency was even more apparent in TNBC patients (HR = 0.22, P = 6.6e−05) (Fig. 1H, right panel).

**Table 1:** Clinical parameters of patients for response evaluation in this study.

Feather	IL-23A^Low^	IL-23A^High^	Number	*P value
All cases	8	14	22	
Gender				
Female	8	14	22	
Age (years)				
< 35	2	0	2	0.121
≥ 35	6	14	20	
BMI				
18.5–< 24	5	5	10	0.352
24–< 28	3	8	11	
≥ 28	0	1	1	
Tumor size (cm)				
< 2	0	1	1	0.383
2–5	8	12	20	
5	0	1	1	
ORR				
PR	2	8	10	0.004
SD	1	5	6	
PD	5	1	6	
TNM stage				
II	6	10	16	0.631
III	2	4	6	

Total data from 22 TNBC patients who underwent neoadjuvant chemotherapy were analyzed. Patients were divided into two groups based on IL-23A expression levels assayed by immunohistochemistry (IHC). The IL-23A^Low^ group comprised 8 patients with an IL-23A IHC score of 0–6, while the IL-23A^High^ group consisted of 14 patients with an IL-23A IHC score of 7–12. Data were analyzed by chi-squared test. *P < 0.05 indicates statistically significant.

### IL-23 and PD-1 mAb synergistically enhance the cytotoxic effect of tumor-cell-specific CTLs in vitro

**Figure 2:**
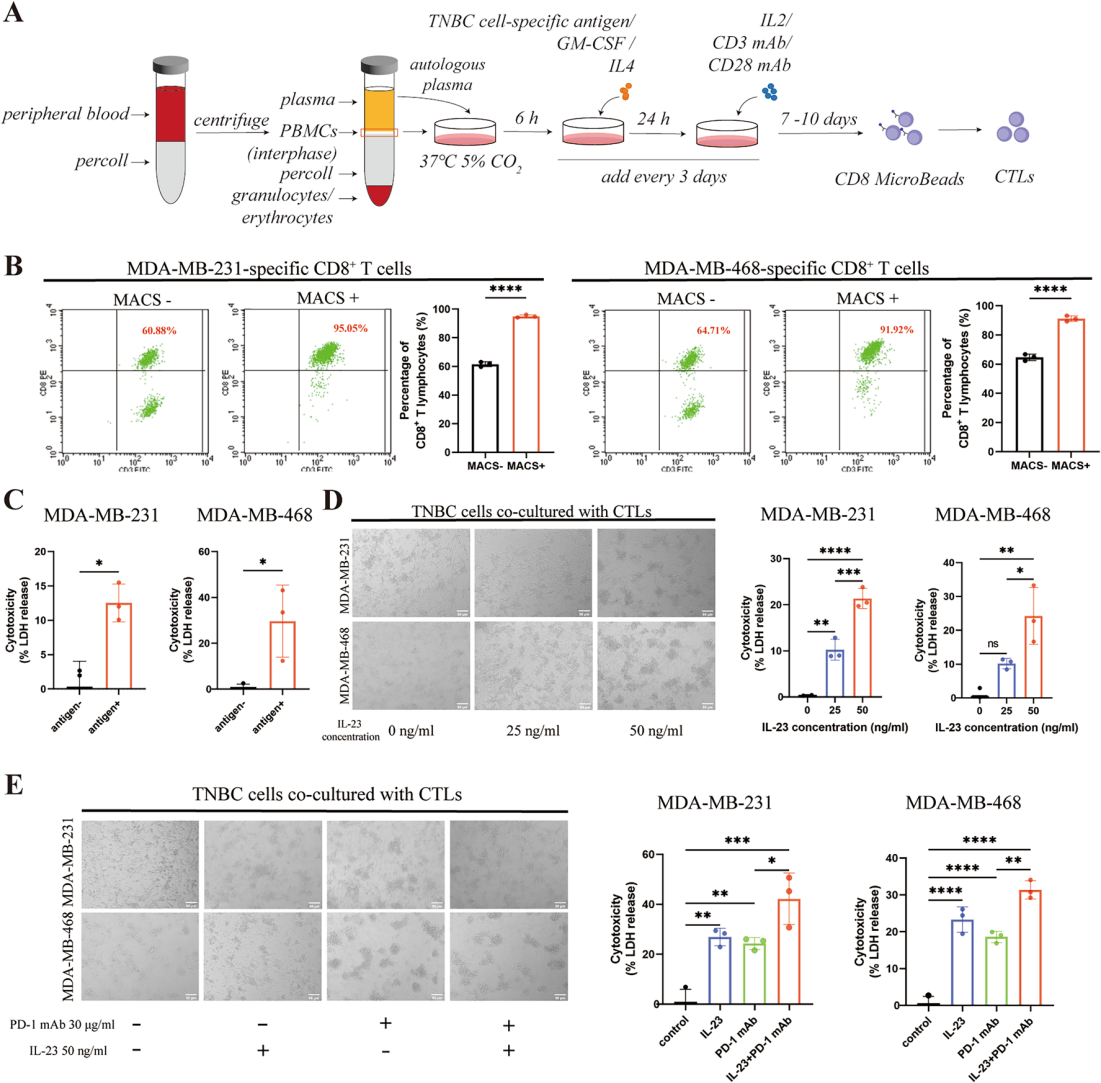
IL-23 and PD-1 mAb synergistically enhance the cytotoxic effect of TNBC cell-specific CTLs in vitro. (**A**) The illustration of the generation and purification of TNBC cell-specific CD8^+^ T lymphocytes. (**B**) Flow cytometry assays detecting the percentages of TNBC cell-specific antigen-induced CD8^+^ T lymphocytes before or after magnetic activated cell sorting (MACS) and the quantifications (n = 3). Left panel, MDA-MB-231-specific T lymphocytes; Right panel, MDA-MB-468-specific T lymphocytes. (**C**) The quantifications of cytotoxicity (quantified by LDH release) on TNBC cells by co-cultured CTLs that were induced with or without specific TNBC cell antigens. Left panel, MDA-MB-231 cell line. Right panel, MDA-MB-468 cell line. (**D**) Left panel, representative morphology of TNBC cells co-cultured with TNBC cell-specific CTLs and treated with 0, 25 ng/ml, 50 ng/ml exogenous IL-23. Scale bars, 50 μm. Right panel, the quantifications of cytotoxicity (quantified by LDH release). (**E**) Left panel, representative morphology of TNBC cells co-cultured with TNBC cell-specific CTLs and treated with control, 50 ng/ml exogenous IL-23, 30 μg/ml PD-1 mAb, and a combination of 50 ng/ml exogenous IL-23 and 30 μg/ml PD-1 mAb. Scale bars 50 μm. Right panel, the quantifications of cytotoxicity (quantified by LDH release). Error bars represent SD, *P < 0.05; **P < 0.01; ***P < 0.001; ****P < 0.0001.

We further investigated how IL23A was involved in anti-tumor cell immunity. PBMCs were activated by whole tumor cell antigens and expanded by cytokines (Fig. 2A). CD8^+^ T lymphocytes (CTLs) were then sorted by MACS and validated through FCM assays (Fig. 2B). We established a co-culture system using a ratio of 20:1 for CD8^+^ T lymphocytes and TNBC cells to explore the cytotoxic effect of CTLs on TNBC cells. Tumor-cell-specific CD8^+^ T lymphocytes exhibited a more potent cytotoxic effect on TNBC cells in vitro compared to CIK cells activated by cytokines, highlighting the specificity of these T cells in targeting and eliminating TNBC cells (Fig. 2C). The cytotoxic effect of tumor-cell-specific CTLs in vitro was significantly promoted by exogenous IL-23 in a dose-dependent manner. Morphological observation corroborated this conclusion and clearly showed the increased cytotoxicity on TNBC cells after treatment of exogenous IL-23 (Fig. 2D). Like IL-23, PD-1 mAb could also enhance the cytotoxic effects of CTLs at an optimal concentration of 30 μg/ml (Supplementary Fig. S2). Interestingly, the combination of exogenous IL-23 and PD-1 mAb synergistically improved the cytotoxic effect of tumor-cell-specific CTLs in vitro (Fig. 2E). We also detected levels of cytotoxic molecules in the supernatant of the co-culture system and observed a synergistic upregulation of Granzyme B and IL-2 by IL-23 and PD-1 mAb. (Supplementary Fig. S3).

### IL-23 and PD-1 mAb synergistically enhance the cytotoxic effect of tumor-cell-specific CTLs in vivo

**Figure 3:**
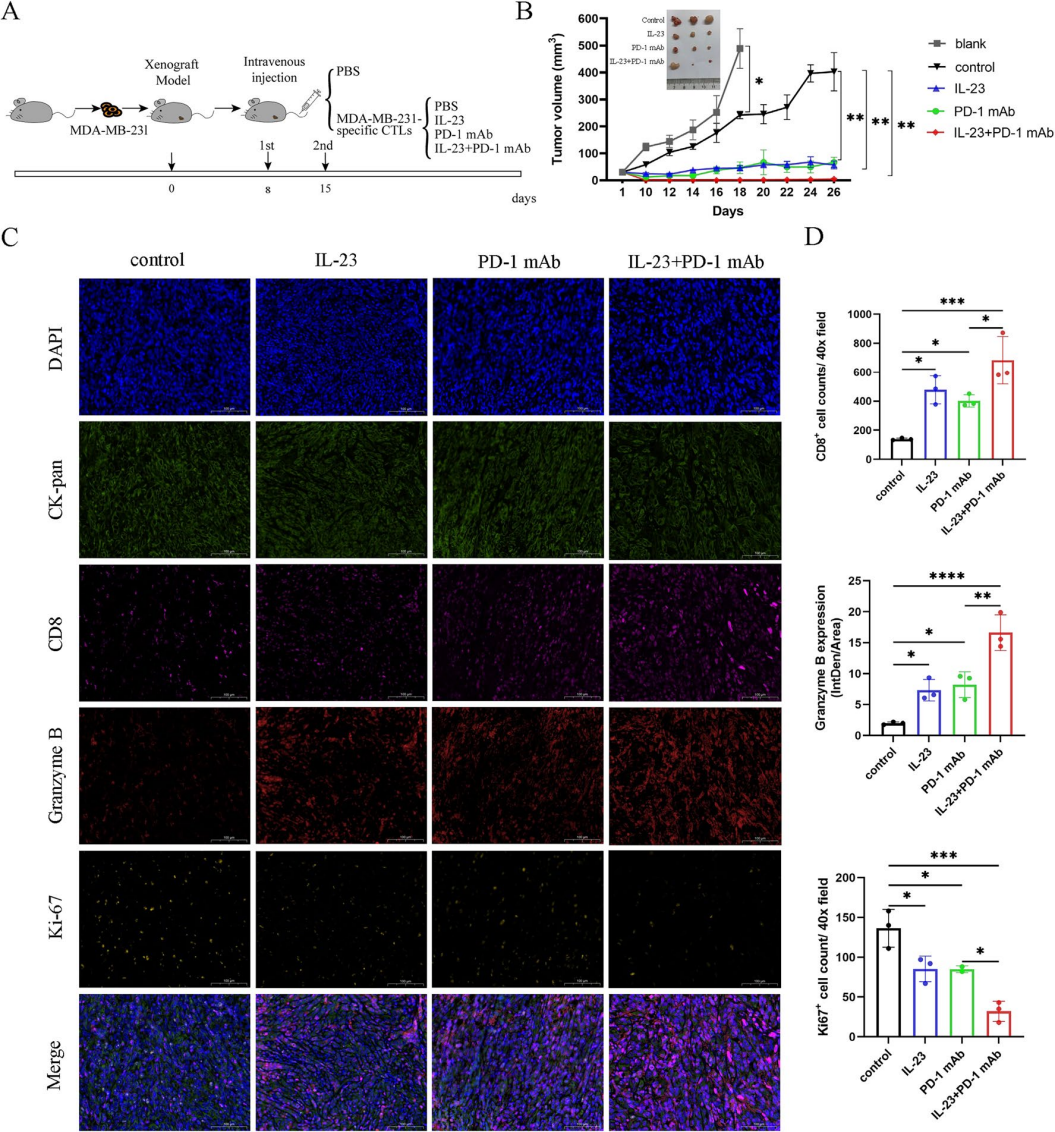
IL-23 and PD-1 mAb synergistically enhance the cytotoxic effect of TNBC cell-specific CTLs in vivo. (**A**) Illustration of the animal study. When the tumor reached approximately 30 mm^3^, PBS control (n = 3–5) or MDA-MB-231-specific CTLs (n = 12–20) were intravenously administrated into the tail vein. Next, mice received administration of CTLs were intravenously treated with PBS control, IL-23, PD-1 mAb, and a combination of IL-23 and PD-1 mAb (n = 3–5 in each group), respectively. The treatment was performed once a week for a total of 2 treatments. Tumor size was measured every two days with a caliper. (**B**) Tumor growth curves of the xenograft tumors. Photographs of tumors from the four groups that received CTLs administration were exhibited. The mice that received only PBS treatment were sacrificed when the difference in tumor volume was significant compared to the other four groups. (**C**) The representative immunofluorescence staining of CK-pan, CD8, Granzyme B, and Ki67 in the xenograft tumors from mice that received CTLs treatment. Scale bars 100 μm. (**D**) The quantifications of infiltration of CD8^+^ T cells, Ki-67 positive cells, and the fluorescence intensity of Granzyme B in the xenograft tumors from mice that received CTLs treatment. Error bars represent SD, *P < 0.05; **P < 0.01; ***P < 0.001; ****P < 0.0001.

Next, the synergistic effects of IL-23 and PD-1 mAb on tumor inhibition were investigated in vivo. The mice receiving tumor-cell-specific CTLs were treated intravenously with IL-23, PD-1 mAb, and a combination of IL-23 and PD-1 mAb with a PBS control group (Fig. 3A). The administration of MDA-MB-231-specific CTLs effectively inhibited tumor growth in vivo. In addition, tumor growth was significantly suppressed in both the IL-23 and the PD-1 mAb groups compared to the CTLs group alone. Moreover, the combined treatment of IL-23 and PD-1 mAb led to a reduction in tumor size compared with other groups (Fig. 3B). These findings suggested that IL-23 and PD-1 mAb could synergistically inhibit tumor growth in vivo. Apart from that, we also evaluated the potential side effects induced by various treatments. Our findings indicated that neither the individual administration of IL-23 and PD-1 mAb nor their combined use resulted in significant toxicity impacting the weight or liver and kidney functions in mice (Supplementary Fig. S4). The results indicated the safety of combined treatment with IL-23 and PD-1 mAb. The mIHC analysis indicated a significant upregulation in the infiltration and activation of CD8^+^ T cells in the tumor microenvironment after treatment of IL-23 and PD-1 mAb. Furthermore, the combination of IL-23 and PD-1 mAb facilitated a more significant increase in CD8^+^ T cell infiltration and Granzyme B expression compared to other groups (Fig. 3C,D).

### PIK3R1 was co-downregulated in CTLs by exogenous IL-23 and PD-1 mAb

**Figure 4:**
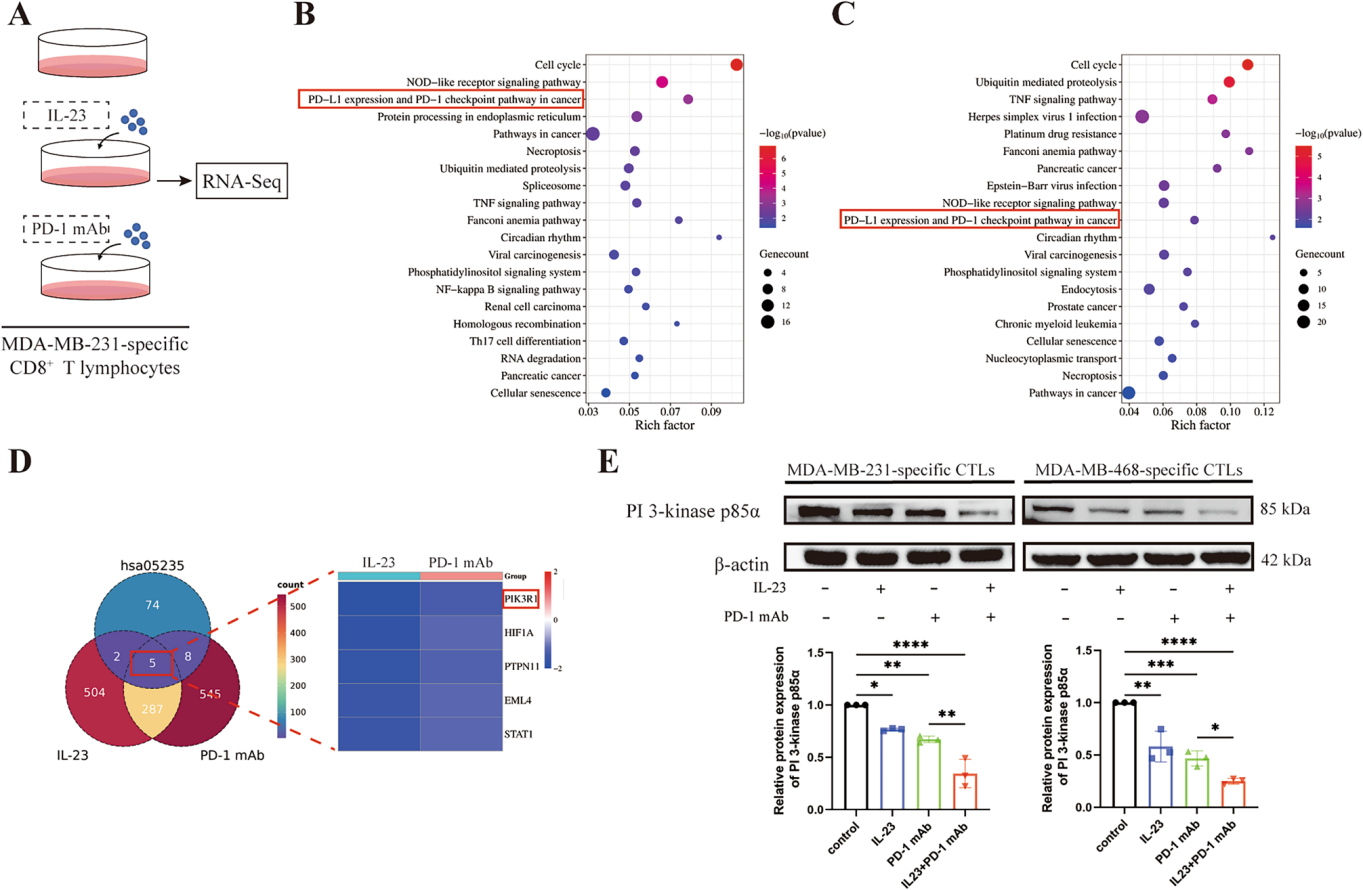
IL-23 and PD-1 mAb synergistically downregulated PIK3R1. (**A**) The illustration of experimental design for RNA sequencing (RNA-seq). MDA-MB-231-specific CTLs treated with control, 50 ng/ml exogenous IL-23, and 30 μg/ml PD-1 mAb were subjected to RNA-seq. (**B**) The KEGG enrichment analysis of RNA-seq results on MDA-MB-231-specific CTLs treated with or without 30 μg/ml PD-1 mAb. (**C**) The KEGG enrichment analysis of RNA-seq results on MDA-MB-231-specific CTLs treated with or without 50 ng/ml exogenous IL-23. The horizontal axis represents gene enrichment, while the vertical axis represents the enriched pathway name. The color scale presents the *p*-value, and the size of the dots indicates the number of genes enriched in particular pathways. (**D**) Left panel, Venn diagram showing the overlapping significantly affected genes regulated by both IL-23 and PD-1 mAb in MDA-MB-231-specific CTLs and genes involved in KEGG hsa05235 pathway (namely, PD-L1 expression and PD-1 checkpoint pathway in cancer). Right panel, the heatmap exhibited the five genes identified in the Left panel, with PIK3R1 being the most downregulated gene. (**E**) Protein expression levels of PI3-kinase p85α in MDA-MB-231- and MDA-MB-468-specific CTLs followin treatment with control, IL-23, PD-1 mAb, and a combination of IL-23 and PD-1 mAb, respectively. Upper panel, the representative images of western blot, adjusted by protein expression levels of β-actin. Lower panel, quantitative analysis of western blot. The PVDF membranes were cropped at 50–70 kDa before antibody incubation. The original blots are presented in Supplementary Figs. S7 and S8. Error bars represent SD, *P < 0.05; **P < 0.01; ***P < 0.001; ****P < 0.0001.

To excavate the potential mechanisms underlying the synergistic effect of IL-23 and PD-1 mAb in anti-tumor immunity, we performed RNA-seq analysis on MDA-MB-231-specific CTLs following the treatment with exogenous IL-23 (50 ng/ml) or PD-1 mAb (30 μg/ml) (Fig. 4A). KEGG enrichment analysis demonstrated a significant alteration of PD-L1 expression and PD-1 checkpoint pathway both in IL-23-treated and PD-1 mAb-treated tumor-cell-specific CTLs (Fig. 4B,C). Among the genes within this pathway, we discovered that Phosphoinositide-3-Kinase Regulatory Subunit 1 (*PIK3R1*) exhibited the most significant alteration, specifically downregulated by both IL-23 and PD-1 mAb treatment alone (Fig. 4D). Consistent with the results of RNA-seq analysis, western blot analysis was conducted and validated the decrease in the protein expression level of PI 3-kinase p85α (coded by *PIK3R1*) following treatment with IL-23 or PD-1 mAb in both MDA-MB-231- and MDA-MB-468-specific CTLs (Fig. 4E). Notably, when IL-23 and PD-1 mAb were combined, we observed a further reduction in p85α expression in TNBC-specific CTLs compared to treatment with PD-1 mAb alone (Fig. 4E), indicating IL-23 and PD-1 mAb had a synergistic effect on reducing p85α expression in TNBC cell-specific CTLs.

### Exogenous IL-23 and PD-1 mAb synergistically enhanced CTLs function via the PI3K-AKT pathway

**Figure 5:**
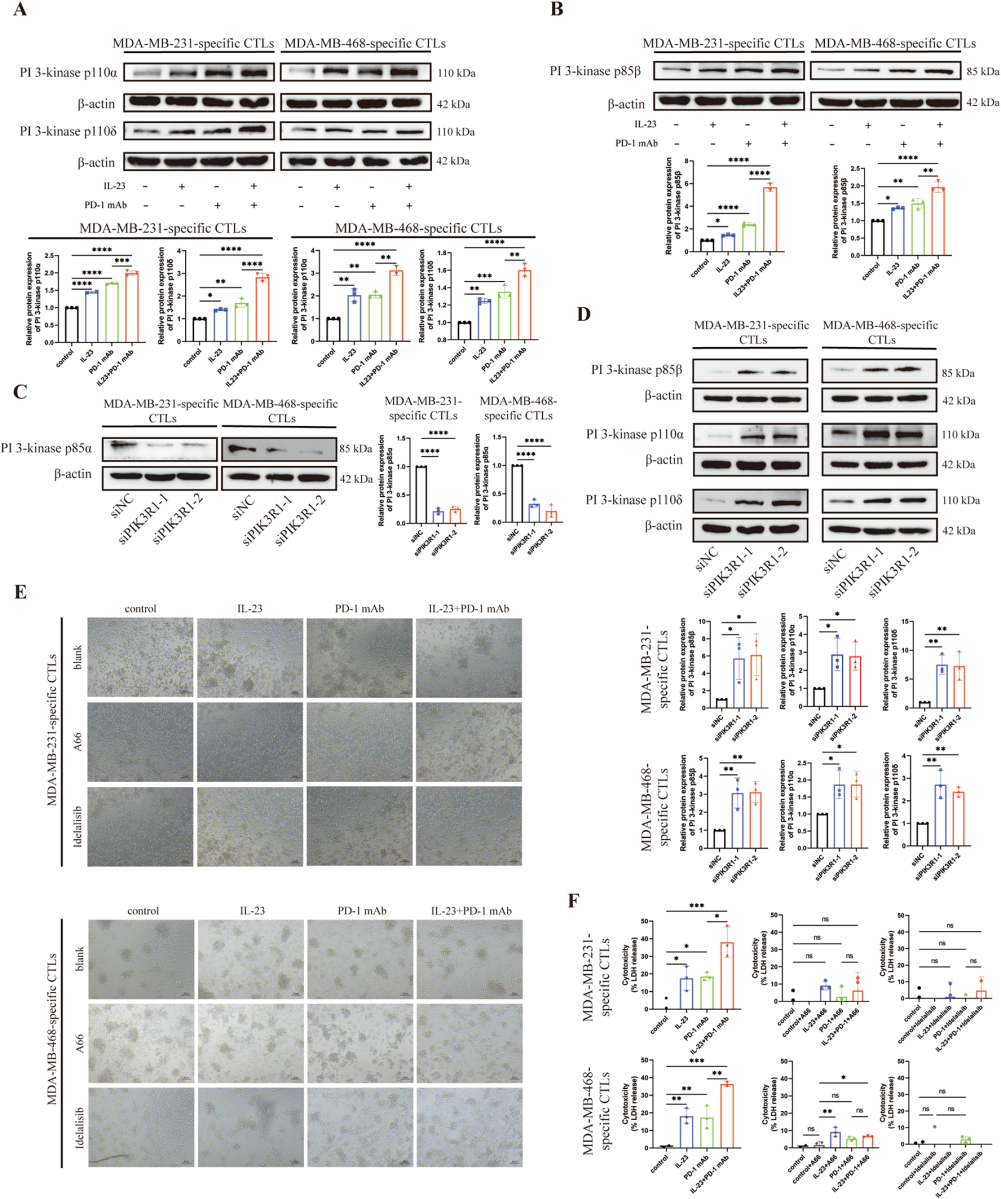
Exogenous IL-23 and PD-1 mAb synergistically enhanced CTLs function via the activation of p110. (**A**) The protein expressions of PI3-kinase p110α and p110δ in MDA-MB-231- and MDA-MB-468-specific CTLs following treatment with control, IL-23, PD-1 mAb, and a combination of IL-23 and PD-1 mAb, respectively. Upper panel, the representative images of western blot, adjusted by protein expression levels of β-actin. Lower panel, quantitative analysis of western blot. The PVDF membranes were cropped at 50–70 kDa before antibody incubation. The original blots are presented in Supplementary Figs. S7–S9. (**B**) The protein expression levels of PI3-kinase p85β in MDA-MB-231- and MDA-MB-468-specific CTLs following treatment with control, IL-23, PD-1 mAb, and a combination of IL-23 and PD-1 mAb, respectively. Upper panel, the representative images of western blot, adjusted by protein expression levels of β-actin. Lower panel, quantitative analysis of western blot. The PVDF membranes were cropped at 50 ~ 70 kDa before antibody incubation. The original blots are presented in Supplementary Figs. S7 and S8. (**C**) The protein expression of PI3-kinase p85α after knockdown of PIK3R1. Left panel, the representative images of western blot, adjusted by protein expression levels of β-actin. Right panel, quantitative analysis of western blot. The PVDF membranes were cropped at 70 kDa before antibody incubation. The original blots are presented in Supplementary Fig. S10. (**D**) The protein expression levels of PI3-kinase p85β, p110α, and p110δ in MDA-MB-231- and MDA-MB-468- specific CTLs following knockdown of PIK3R1. Upper panel, the representative images of western blot, adjusted by protein expression levels of β-actin. Lower panel, quantitative analysis of western blot. The PVDF membranes were cropped at 70 kDa before antibody incubation. The original blots are presented in Supplementary Figs. S10–S12. (**E**) The representative morphology of MDA-MB-231 and MDA-MB-468 cells co-cultured with specific CTLs following treatment with control, IL-23, PD-1 mAb, and a combination of IL-23 and PD-1 mAb, with or without treatment with A66 (100 nM) and Idelalisib (10 nM). Upper panel, the MDA-MB-231 specific co-culture systems. Lower panel, the MDA-MB-468 specific co-culture systems. Scale bars 50 μm. (**F**) The quantifications of cytotoxicity (quantified by LDH release). Upper panel, the MDA-MB-231 specific CTLs. Lower panel, the MDA-MB-468 specific CTLs. Error bars represent SD, *P < 0.05; **P < 0.01; ***P < 0.001; ****P < 0.0001.

*PIK3R1* encodes the regulatory subunit p85α of class IA Phosphatidylinositol 3-kinase (PI3Ks), which forms heterodimers with one of the catalytic subunits p110α, p110β, or p110δ. The repressive regulation of p110 by p85α have been demonstrated in a domain-specific manner^24^. We hypothesized that downregulation of p85α in TNBC-specific CTLs would induce increased activation of p110. Consistent with our hypothesis, expression levels of both p110α and p110δ were elevated by treatment with IL-23 and PD-1 mAb individually, and IL-23 could further enhance expression of p110α and p110δ when combined with PD-1 mAb compared to individual treatment with PD-1 mAb (Fig. 5A). P85β is another regulatory subunit of p110 and is believed to be associated with a reduced inhibition of p110^25^. Notably, the regulation of p85β by IL-23, PD-1 mAb, and a combination with IL-23 and PD-1 mAb was similar to that of p110 (Fig. 5B). To further explore the regulation of p85β and p110 in TNBC-specific CTLs, *PIK3R1* was knocked down with decreased expression of p85α (Fig. 5C). This knockdown led to increased expression of p85β, p110α, and p110δ, indicating the negative regulation of p110 by p85α in TNBC-specific CTLs (Fig. 5D).

We further investigated which isoform of p110 contributes to the anti-tumor immunity of TNBC-specific CTLs. We employed the p110α selective inhibitor A66 and p110δ selective inhibitor idelalisib to specifically block the activity of p110α or p110δ, respectively. The LDH assay was performed to access the cytotoxic effects in the co-culture system comprising TNBC cells and the corresponding TNBC-specific CTLs. The cytotoxic effects of the CTLs were strongly inhibited by A66. However, the increased cytotoxic effects induced by IL-23 and the combined treatment with IL-23 and PD-1 mAb were still observed. Notably, inhibition of p110δ completely eliminated the enhanced cytotoxic effects induced by IL-23, PD-1 mAb, and combined treatment with IL-23 and PD-1 mAb, suggesting that p110δ is the predominant p110 isoform contributing to the cytotoxic effects of CTLs (Fig. 5E,F).

**Figure 6:**
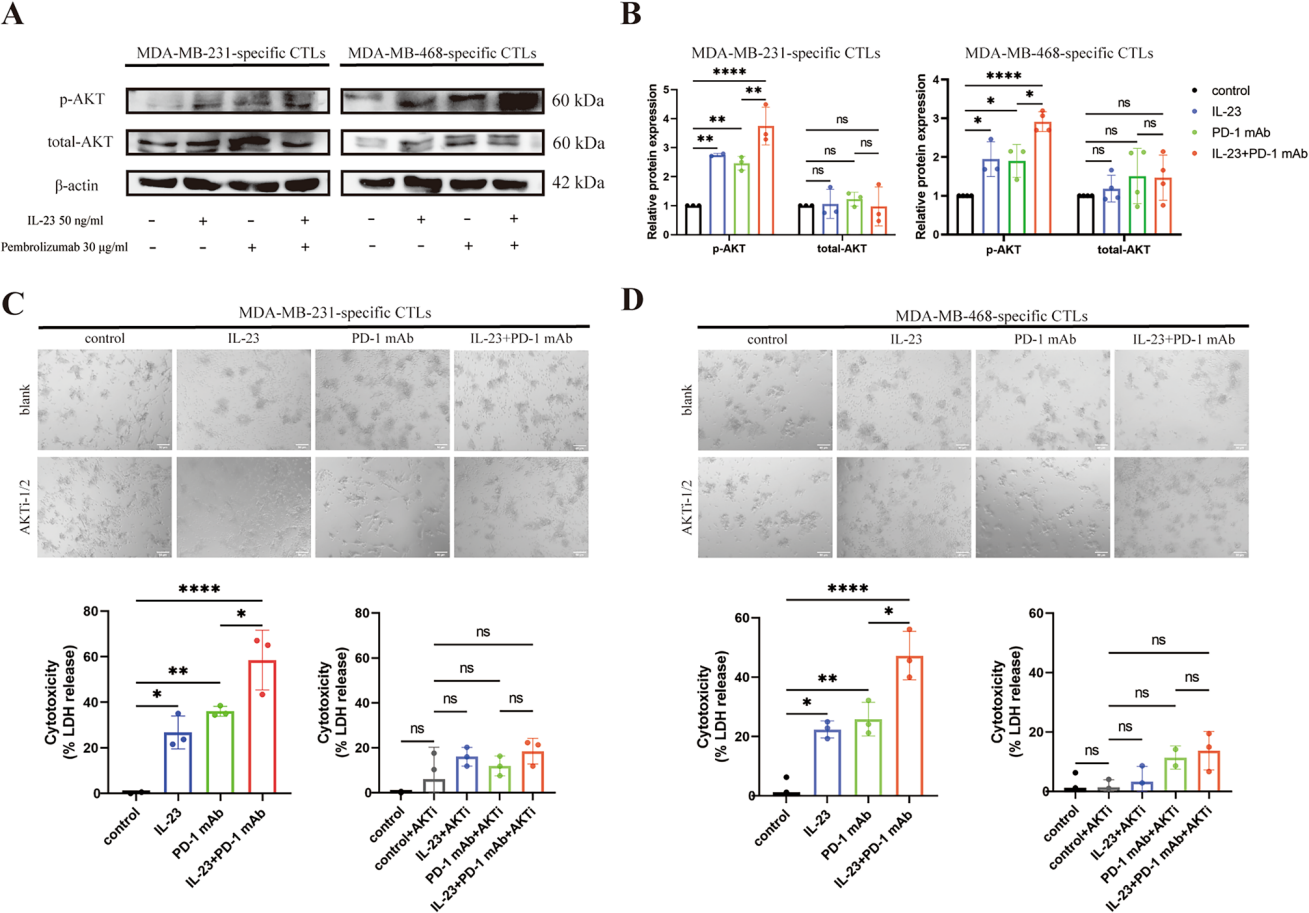
IL-23 and PD-1 mAb enhance TNBC cell-specific CTLs' cytotoxic effect by co-activating the AKT pathway. (**A**) Protein expression levels of total-AKT and phospho-AKT in TNBC cell-specific CTLs following treatment with control, IL-23, PD-1 mAb, and a combination of IL-23 and PD-1 mAb, respectively, detected by western blot, adjusted by protein expression levels of β-actin. Left panel, MDA-MB-231-specific CTLs; Right panel, MDA-MB-468-specific CTLs. The PVDF membranes were cropped at 70 kDa and 50 kDa before antibody incubation. The original blots are presented in Supplementary Figs. S13 and S14. (**B**) Quantitative analysis of western blot. (**C**) Upper panel, representative morphology of MDA-MB-231 cells co-cultured with MBA-MB-231-specific CTLs following treatment with control, IL-23, PD-1 mAb, and a combination of IL-23 and PD-1 mAb, with or without AKT inhibitor (AKTi-1/2). Lower panel, the quantifications of cytotoxicity (quantified by LDH release). (**D**) Upper panel, representative morphology of MDA-MB-468 cells co-cultured with MBA-MB-468-specific CTLs following treatment with control, IL-23, PD-1 mAb, and a combination of IL-23 and PD-1 mAb, with or without AKTi-1/2. Lower panel, the quantifications of cytotoxicity (quantified by LDH release). Error bars represent SD, *P < 0.05; **P < 0.01; ****P < 0.0001.

PI3Ks function to catalyze the production of phosphatidylinositol-3,4,5-triphosphate (PIP3), which can bind to and activate downstream proteins via a PH domain. The Akt pathway, known to play a critical role in immune responses, is a significant downstream target of the PI3K pathway. Notably, following the treatment with p110α selective inhibitor A66 or p110δ selective inhibitor idelalisib, we observed a reduction in the phosphorylation levels of AKT in TNBC-specific CTLs, indicating the contributory roles of p110α and p110δ in the activation of AKT pathway (Supplementary Fig. S5). Here, we also examined the expression levels of total-AKT and phospho-AKT in TNBC-specific CTLs following the treatment with IL-23 and PD-1 mAb. We observed an increase in AKT phosphorylation in response to treatment with either IL-23 or PD-1 mAb. Intriguingly, the IL-23 and PD-1 mAb combination exhibited an even more pronounced enhancement of AKT phosphorylation than either agent alone (Fig. 6A,B). However, the expression of pan-AKT did not display the same pattern.

Next, we employed the AKT inhibitor III (AKTi-1/2) to inhibit the activity of phosphorylated AKT. IL-23 and PD-1 mAb were introduced into the co-culture system consisting of TNBC cells and the corresponding TNBC-specific CTLs. In line with our previous results, IL-23 and PD-1 mAb demonstrated a synergistic effect on anti-tumor immunity. However, those effects were abrogated when AKTi-1/2 was additionally administered (Fig. 6C,D), indicating that AKT phosphorylation may contribute to the enhanced cytotoxic effect of CTLs mediated by IL-23 and PD-1 mAb.

**Figure 7:**
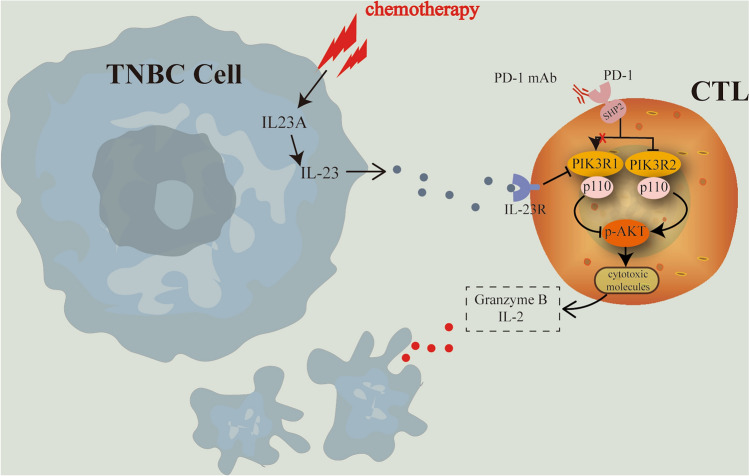
Schematic diagram of the molecular mechanisms demonstrated in this article. IL23A was upregulated by chemotherapy in TNBC cells. Subsequently, TNBC cell-derived IL-23 interacts with IL23R, which is expressed on the surface of activated lymphocytes. The IL-23/IL23R axis participates in the downstream signaling to downregulate PIK3R1, facilitating the activation of the PI3K-AKT pathway and the subsequent upregulation of cytotoxic molecules such as Granzyme B and IL-2. PD-1 mAb is also involved in the downregulation of PIK3R1 and plays a synergistic role with IL-23 in enhancing the cytotoxic effect of CTLs in anti-tumor immunity.

Altogether, the combination of IL-23 and PD-1 mAb could intensively inhibit *PIK3R1* which encodes the regulatory subunit p85α of class IA PI3Ks. Subsequently, the catalytic subunits p110α and p110δ were upregulated and significantly contribute to the enhanced cytotoxic effects of TNBC-specific CTLs, with p110δ being the predominant contributor. The phosphorylation of AKT, a critical downstream pathway of PI3Ks closely associated with immune responses, plays a crucial role in this process. (Fig. 7).

## Discussion

Triple-negative breast cancer (TNBC) exhibits several unique biological characteristics compared to other subtypes of breast cancer, including more immunogenic with lymphocyte infiltrating in the tumor microenvironment. The tumor-infiltrating lymphocytes (TILs), believed to be closely associated with a favorable prognosis, were more abundant in TNBC. Additionally, expression of PD-L1 driven by loss of PTEN was noticed to increase in TNBC, indicating the promising potential of immunotherapy. The FDA has already approved some immune checkpoint inhibitors (ICIs) in combination with chemotherapeutic drugs to treat TNBC^26,27^. However, the clinical results from different combination strategies of chemotherapy regimens and PD-1/PD-L1 mAb were significantly different^10,13–15,28^. To date, there are three problems we are facing when using ICIs to treat TNBC. First, how do we select proper patients who will benefit from ICIs? Second, how do we arrange the combination of ICIs and chemotherapy? Third, how do the chemotherapy and ICIs synergistically inhibit the tumor growth of TNBC? Exploring the detailed mechanism of cross-effect between chemotherapy and ICIs could supply a more reasonable way of using the two anti-tumor modalities.

It has been widely reported that chemotherapy could utilize two ways to enhance anti-tumor immunotherapy: promoting immunogenic tumor cell death or modulating the immunosuppressive tumor microenvironment. Importantly, we identified a new way in this study. IL-23 is a heterodimeric cytokine constructed by two subunits, p19 (coded by *IL23A*) and p40 (coded by *IL12B*). It is produced by activated dendritic cells and macrophages and regulates lymphocyte activation and differentiation^29–31^. In the present study, we found that the expression of IL23A was upregulated in TNBC cells by chemotherapeutics, validated by both in vitro models and resected tumor tissues from TNBC patients. Moreover, we confirmed the effects of IL-23 on the activation of tumor-cell-specific CTLs, which are also the direct target of ICIs. Our findings proposed a potential molecular link between chemotherapy and ICIs that could synergistically activate the anti-tumor immunotherapy.

IL-23 belongs to the IL-12 cytokine family and shares subunit p40 with IL-12. IL-23 functions by interacting with its receptor IL23R and activating downstream signaling pathways and is involved in regulating immune responses, particularly in the proliferation and stability of Th17 cells^32,33^. IL23R is expressed only after activation of T cells and creates a positive feedback loop with IL-23^30^. Notably, the IL-23/IL23R axis was reported to foster the expansion of activated T cells and enhance the anti-tumor immunity in solid tumors^34^. In our research, increased expression of IL23A was observed in both TNBC cells treated with chemotherapeutic drugs and resected tumor tissues from patients who underwent neoadjuvant chemotherapy. Moreover, IL23A was a favorable indicator for the objective response rate (ORR) in TNBC patients who received neoadjuvant chemotherapy. Subsequently, we investigated the regulatory role of IL-23 in anti-tumor immunity and found that IL-23 could enhance anti-tumor immunity both in vitro and in vivo. In addition, we discovered that IL-23 and PD-1 mAb had a synergistic effect in improving anti-tumor immune response, which was associated with increased levels of cytotoxic molecules such as Granzyme B and IL-2. The synergistic role of IL-23 and PD-1 mAb may partially explain the molecular mechanisms underlying the combined treatment of chemotherapy and anti-PD-1 therapy.

In our recent work^35^, we found that IL23 could enhance the immunotherapy efficacy of lung adenocarcinoma by activating the IL-9 autocrine loop of CTLs. However, CTLs of TNBC patients might take another pathway for activation. The KEGG enrichment analysis of RNA sequencing results on TNBC-specific CTLs indicated that PD-L1 expression and PD-1 checkpoint pathway in cancer was co-inhibited by IL23 and PD-1 mAb through downregulating *PIK3R1*, which encodes the regulatory subunit p85α of class IA PI3Ks.

The activity of phosphoinositide 3-kinase (PI3K) plays a crucial role in various cellular processes such as metabolism, survival, and growth^36^. PI3Ks catalyze the production of phosphatidylinositol-3,4,5-triphosphate (PIP3), a lipid second messenger that binds to and activates downstream molecules via the PH domain. Of note, the Akt pathway, which is tightly linked to immune responses through the regulation of mTOR and Foxo transcription factors, have been identified as a critical downstream component of PI3Ks, thus garnering significant attention^37,38^. *PIK3R1* encodes P85α which is the regulatory subunit of class IA PI3Ks and elicits inhibitory effects on the catalytic subunits of class IA PI3Ks via a domain-specific manner^24,25,39^. Specifically, the interaction between p85α nSH2 domain and p110α helical domain, as well as the interaction between p85α iSH2 domain and p110α C2 domain, can inhibit p110α activity. In this study, we identified *PIK3R1* as the common downstream molecule down-regulated by IL-23 and PD-1 mAb in TNBC-specific CTLs, accompanied by decreased expression of p85α and increased expression of p110α and p110δ. In addition, knockdown of *PIK3R1* in TNBC-specific CTLs led to a significant upregulation of p110α and p110δ, indicating an inhibitory effect of p85α on PI3K pathway. Consistent with our hypothesis, the phosphorylation of AKT was synergistically enhanced by IL-23 and PD-1 mAb. To further elucidate the role of the PI3K-AKT pathways in the regulation of cytotoxic effects of CTLs, we employed the selective inhibitor of p110α, p110δ, and AKT, and assessed their impact on the cytotoxic effects of TNBC-specific CTLs. Our findings revealed that p110δ was the predominant catalytic subunit contributing to the CTLs cytotoxic effects triggered by IL-23 and PD-1 mAb, with AKT phosphorylation playing a critical role in this mechanism. The increased expression of p85β was detected after knockdown of PIK3R1. P85β, coded by PIK3R2, is another regulatory subunit of PI3Ks. It has been reported that p85β plays a role in T cell differentiation and maturation^40^. Mechanically, p85β is associated with active PI3K signaling. A plausible explanation is that p85β acts as a less effective inhibitor of the PI3K catalytic subunit, resulting in diminished inhibition of p110^25^. The phosphorylation of AKT has been identified as a downstream pathway of PI3K and contribute to the enhanced cytotoxic effects of CTLs. In the inactive state of AKT, the PH domain acts as an inhibitory element, and the interaction between PH domain and PI3K lipid products contributes to phosphorylation and activation of AKT^41,42^. Besides class IA PI3Ks mentioned in our study, other proteins may also contribute to the activation of AKT, such as class II PI3K-C2α and CK2^43,44^. The involvement of these proteins in regulating the immune responses warrants further investigation.

In conclusion, we found that IL-23, an immunological cytokine upregulated by chemotherapy in TNBC cells, plays a vital role in enhancing the anti-tumor immune response of cytotoxic T cells (CTLs), especially in combination with PD-1 mAb. Our findings could explain the synergistic effects between chemotherapy and ICIs. They might provide a molecular marker that could be used to predict the effects of combination chemotherapy therapy and PD-1 mAb in TNBC.

## Methods

### Clinical samples

Resected tumor tissue sections were obtained from patients pathologically diagnosed with TNBC (all female) who underwent surgical treatment at the Department of General Surgery, Affiliated Jinling Hospital, Medical School of Nanjing University (Nanjing, China) from June 2016 to June 2022, with all the patients providing informed written consent. In this cohort, 27 patients received neoadjuvant chemotherapy, while 37 patients (controls) did not. Patients whose data was incomplete were excluded for clinical response evaluation. Peripheral blood was obtained from healthy donors for isolation of CD8^+^ T lymphocytes. All participants provided informed written consent. The study was approved by the Institutional Ethics Committee of Jinling Hospital (DZQH-KYLLFs-23-31) and conducted following the Declaration of Helsinki and government policies.

### Tumor cell lines

The human triple-negative breast cancer cell lines MDA-MB-231 and MDA-MB-468 were obtained from the Shanghai Institutes for Biological Sciences, Chinese Academy of Sciences (Shanghai, China), and cultured in L15 medium (Gibco, USA) supplemented with 10% fetal bovine serum (FBS; ExCell Bio, Uruguay) and 1% penicillin/streptomycin (Gibco, USA) at 37 °C without CO_2_ equilibration.

### Isolation and culture of tumor-specific CTLs

Percoll density gradients (60%, MilliporeSigma, Germany) were used to isolate peripheral blood mononuclear cells (PBMCs) from the blood of healthy donors. The isolated PBMCs were resuspended with RPMI-1640 medium (10% autologous serum) at a density of 1 × 10^6^ /ml and implanted in a 10 cm dish. Six hours later, TNBC cell-specific antigens (prepared by repeatedly freezing and thawing TNBC cells three times), recombinant human GM-CSF (PeproTech, USA), and recombinant human IL-4 (PeproTech, USA) were added into the medium. Twenty-four hours later, recombinant human IL-2 (PeproTech, USA), CD3 mAb (OKT3, Thermo Fisher, USA), and CD28 mAb (Thermo Fisher, USA) were added into the medium. The additives were supplemented every three days. Nearly 7–10 days later, tumor-specific CD8^+^ T cells were obtained through magnetic activated cell sorting (MACS) following the manufacturer’s instructions (Miltenyi Biotec, Germany).

### Enzyme-linked immunosorbent assay (ELISA)

Human Granzyme B Quantikine ELISA Kit (R&D Systems, USA) and Human IL-2 Quantikine ELISA kit (R&D Systems, USA) were used to measure the secretion of Granzyme B and IL-2 according to the manufacturer’s instructions.

### Immunohistochemistry (IHC)

Tumor samples were deparaffinized and rehydrated according to standard protocols. Heat-induced antigen retrieval method (HIER) with citrate buffer (pH = 6.0) was employed for antigen retrieval. Hydrogen peroxide (3%) was used to wipe out the interference from endogenous peroxidase. After blocking with 1% BSA (Beyotime, China), the slides were incubated with primary antibodies [IL-23A (1:500; Proteintech, China), CD8α (1:200; Abcam, UK)] overnight at 4 °C and then the corresponding secondary antibody (ZSGB-BIO, China) for 30 min at room temperature. Afterwards, the slides were subjected to DAB chromogen development (Beyotime, China). The IHC score was measured according to the immunostaining intensity and positive cell distribution. Briefly, the intensity of immunostaining was estimated as follows: 0, negative; 1, weak; 2, moderate; 3, intense. The distribution of positive cells was allotted to five categories: 0, < 5%; 1, 5–25%; 2, 25–50%; 3, 50–75%; 4, > 75%. The overall staining of IL-23A and CD8 was measured by multiplying the intensity score and the score of positive cell distribution.

### Multiplex immunofluorescence staining (mIHC) analysis

Antigen retrieval was performed using heat-induced antigen retrieval (HIER) with citrate buffer (pH = 6.0). Each section was stained successively in several rounds. Each round included endogenous peroxidase blocking with 3% hydrogen peroxide and non-specific site blocking with 1% BSA (Beyotime, China), as well as treatment with the primary antibody [CD8α (1:200; Abcam, UK), pan Cytokeratin (1:200; Abcam, UK), Granzyme B (1:200; Santa Cruz, USA), and Ki-67(1:200; Santa Cruz, USA)] and corresponding secondary horseradish peroxidase-conjugated polymer (ZSGB Bio, China) which was able to amplify tyramide signal to promote the covalent binding of different fluorophores. Finally, the tumor sections underwent nuclear staining with DAPI (Thermo Fisher, USA) and high-resolution fluorescence scanning. The detailed information of reagents and antibodies is listed in Supplementary Table S1. The positive cell number with specific fluorescence and the fluorescence intensity were analyzed by Image J (http://imagej.nih.gov/ij/^45^.

### Flow cytometry (FCM)

Cells for FCM analysis were washed and resuspended at the concentration of 1 × 10^6^/ml. The BD Multi-test CD3 FITC/CD8 PE/CD45 PerCP/CD4 APC reagent (BD Biosciences, USA) was used following the manufacturer’s instructions to identify and determine the percentages of CD8^+^ T lymphocytes.

### Western blot analysis

Protein lysates were collected in RIPA lysis Buffer (Beyotime, China) containing protease and phosphatase inhibitor Cocktail (1:100, New Cell & Molecular Biotech Co., Ltd). Proteins were separated by SDS-PAGE and electro-transferred to PVDF membranes (Millipore, USA), which were blocked with 5% defatted milk or 5% BSA at room temperature for 1 h and incubated with primary antibodies [β-actin (1:20,000; Proteintech, China), IL-23 (1:500; Santa Cruz, USA), PI 3-kinase p85α (1:500; Santa Cruz, USA), PI 3-kinase p85β (1:500; Santa Cruz, USA), PI 3-kinase p110α (1:1000; Proteintech, China), PI 3-kinase p110β (1:500; Santa Cruz, USA), PI 3-kinase p110δ (1:500; Santa Cruz, USA), phosphor-Akt (Ser473) (1:1000; Cell Signaling Technology, USA), AKT (1:5000; Proteintech, China)] at 4 °C overnight. The detailed information of antibodies is listed in Supplementary Table S1. The PVDF membranes were cropped prior to hybridization with the antibodies. The original images which could be put together for full-length blots were provided in the supplementary materials S1. The target proteins were then incubated with anti-rabbit/mouse secondary antibodies (1:1000, CST, USA) at room temperature for 1 h and detected by ECL chemiluminescence (NCM, China) and Clinx ChemiScope imaging system (Clinx, China). Western blots were quantified by Image J (http://imagej.nih.gov/ij/).

### RNA interference

The siRNAs targeting human PIK3R1 were obtained from GenePharma Co. Ltd (Shanghai, China). The sequences for human PIK3R1 siRNA are as follows: siPIK3R1-1: sense (GGAUCAAGUUGUCAAAGAATT), antisense (UUCUUUGACAACUUGAUCCTT); siPIK3R1-2: sense (CAGCUAUUGAAGCAUUUAATT), antisense (UUAAAUGCUUCAAUAGCUGTT), with a negative control: sense (UUCUCCGAACGUGUCACGUTT), antisense (ACGUGACACGUUCGGAGAATT). Transfections were carried out using the siRNA-mate plus reagent following the manufacturer’s instructions.

### Quantitative real-time PCR (qRT-PCR)

TRIzol Reagent (Thermo Fisher, USA) extracted the total RNA of tumor cells. 1ug of total RNA was reverse-transcribed into cDNA using the PrimeScript RT reagent Kit (TaKaRa, Japan). Quantitative Real-time PCR (qPCR) was performed using SYBR Green Master Mix (Thermo Fisher, USA) on a QuantStudio 1 Real-Time PCR System (Thermo Fisher, USA). ACTB was used as an internal control. Expression of genes was quantitated by the 2^-ΔΔ^ CT method. The primer sequences used are IL23A Forward: 5′-GCCTTCTCTGCTCCCTGATA-3′; Reverse: 5′-CTGCTGCCTTTAGGGACTCA-3′. IL12B Forward: 5′-CGGTCATCTGCCGCAA-3′; Reverse: 5′-AACCTAACTGCAGGGCACAG-3′. ACTB: Forward: 5′-CACCATTGGCAATGAGCGGTTC-3′, Reverse: 5′-AGGTCTTTGCGGATGTCCACGT-3′.

### Lactic dehydrogenase (LDH) release assay

Human TNBC cells were seeded into 96-well plates with 5 × 10^3^ cells/well. TNBC cell-specific CTLs (20:1) were added into the medium. After treatment with different drugs for 24 h, the 96-well was centrifuged, and supernatants were collected for cytotoxicity analysis using the Lactic Dehydrogenase Release Assay Kit (Beyotime, China). The absorbance was measured at 490 nm by a Bio-Rad Microplate Reader (Bio-Rad, USA).

### RNA sequencing

Total RNA extracted from MDA-MB-231 cells and MDA-MB-468 cells treated with PBS, 1.25 μg/ml cisplatin, 2.5 μg/ml cisplatin, 7.5 ng/ml paclitaxel, and 15 ng/ml paclitaxel respectively were collected for whole transcriptomic sequencing. Total RNA extracted from tumor-specific CD8^+^ T lymphocytes treated with PBS, 50 ng/ml IL-23, and 30 μg/ml PD-1 mAb separately were collected for mRNA sequencing. The RNA sequencing was performed on an Illumina Novaseq^TM^6000 platform.

### Animal models

Female BALB/c nude mice (CByJ.Cg-*Foxn1*^nu^/J) aged 4–6 weeks were obtained from the Model Animal Research Center of Nanjing University (Nanjing, China) and raised under specific pathogen-free conditions with a 12-h light/dark cycle at 25 ± 2 °C. The animal studies were approved by the Institutional Animal Care and Use Committee of Jinling Hospital, Medical School of Nanjing University (Nanjing, China) and performed following the Public Health Service Policy on Humane Care and Use of Laboratory Animals. The study was reported in accordance with ARRIVE guidelines^46^.

Breast cancer xenograft models were established by implanting MDA-MB-231 cells in the 4th mammary fat pad of BALB/c nude mice (5 × 10^6^ cells per mouse). When the tumor volume reached approximately 30 mm^3^, the mice were randomly divided into five groups, each consisting of 3–5 mice. One group was intravenously injected with phosphate buffer saline (PBS), while the other four were intravenously injected with tumor-cell-specific CTLs. Then, the mice that received CTLs were further injected intravenously with PBS, IL-23, PD-1 mAb, and a combination of IL-23 and PD-1 mAb, respectively. The treatment was performed once a week for a total of 2 treatments. Tumor size was measured every two days with vernier caliper. The tumor volume was calculated using the following formula: (L × W^2^)/2, where L is the longest diameter of the tumor and W is the perpendicular shortest diameter. Finally, the mice were euthanized by cervical dislocation under anesthesia with isoflurane.

### Statistical analysis

One-way ANOVA analysis of difference was used to compare multiple groups, followed by Student’s post hoc two-tailed t-test. Student’s unpaired two-tailed tests were used for comparisons between two groups. Spearman correlation analysis was utilized to analyze correlations. A *P*-value < 0.05 was considered statistically significant. The synergism of two treatments was assessed according to the following formula: q = E_A+B_/[E_A_ + (1 − E_A_) E_B_], where E_A+B_ represents the inhibition rate of combined treatment of IL-23 and PD-1 mAb, E_A_ represents the inhibition rate of IL-23 treatment, and E_B_ represents the inhibition rate of PD-1 mAb treatment (q < 0.85, antagonism; q > 1.15, synergism; 0.85 ≤ q ≤ 1.15, additive)^47^.The analysis was performed by Prism version 9.0.0 software (GraphPad Software).

### Ethics approval

The study was approved by the Institutional Ethics Committee of Jinling Hospital (DZQH-KYLLFS-23-31). The animal studies were approved by the Institutional Animal Care and Use Committee of Jinling Hospital, Medical School of Nanjing University (Nanjing, China) and performed following the Public Health Service Policy on Humane Care and Use of Laboratory Animal.

### Supplementary Information

Supplementary Information.

## Data Availability

The datasets used and/or analysed during the current study are available from the corresponding author on reasonable request.
