# Supplementary material for: Chemotherapy-induced high expression of IL23A enhances efficacy of anti-PD-1 therapy in TNBC by co-activating the PI3K-AKT signaling pathway of CTLs
Source: Sci Rep. 2024 Jun 20;14:14248. doi: 10.1038/s41598-024-65129-7 (PMC11189934; doi:10.1038/s41598-024-65129-7)
Supplement: Supplementary file 1 — Supplementary Information. [file 41598_2024_65129_MOESM1_ESM.pdf]

Chemotherapy-induced high expression of IL23A enhances efficacy of anti-PD-1 therapy in TNBC by co-activating the PI3K-AKT signaling pathway of CTLs

[#]Fan Pan, [#]Jiajing Liu, Ying Chen, Binghan Zhu, Weiwei Chen, Yuchen Yang, Chunyan Zhu, Hua Zhao, Xiaobei Liu, Yichen Xu, Xiaofan Xu, Liqun Huo, [*]Li Xie, [*]Rui Wang, [*]Jun Gu, [*]Guichun Huang

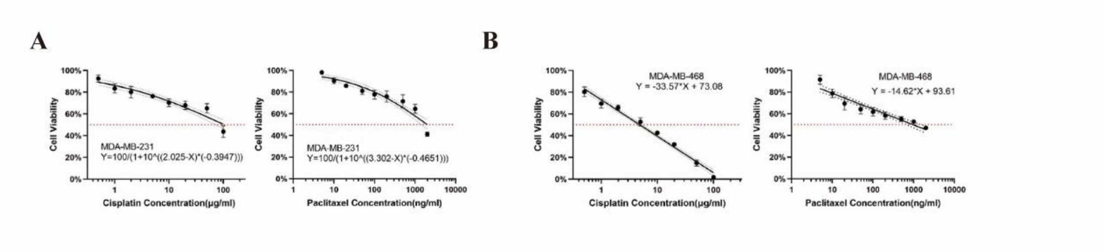

Supplementary Figure 1. The dose-response curve for cisplatin and paclitaxel on MDA-MB-231 and MDA-MB-468 cell lines. (A) The dose-response curve for cisplatin (Left) and paclitaxel (Right) on MDA-MB-231 cells. (B) The dose-response curve for cisplatin (Left) and paclitaxel (Right) on MDA-MB-468 cells.

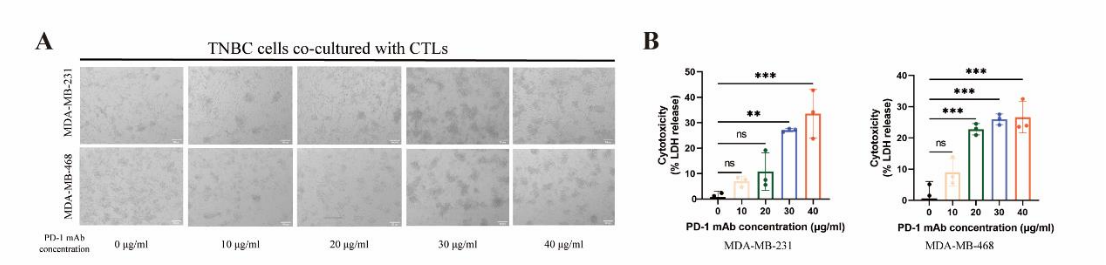

Supplementary Figure 2. PD-1 mAb enhances the cytotoxic effect of TNBC cell-specific CTLs *in vitro*. (A) Representative morphology of TNBC cells co-cultured with TNBC cell-specific CTLs that were treated with 0, 10 μg/ml, 20 μg/ml, 30 μg/ml, and 40 μg/ml PD-1 mAb respectively. Scale bars, 50 μm. (B) The quantifications of cytotoxicity (quantified by LDH release). Left panel, MDA-MB-231 cell line; Right panel, MDA-MB-468 cell line. Error bars represent SD, **, $P<0.01$; ***, $P<0.001$.

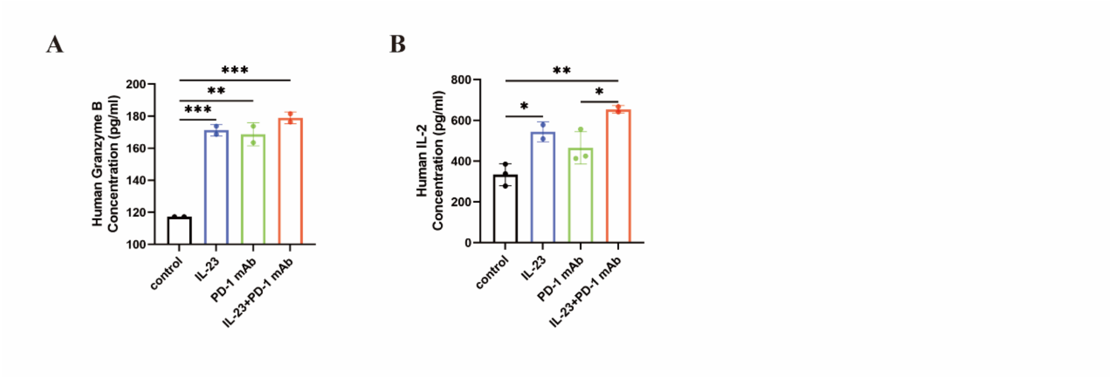

Supplementary Figure 3. IL-23 and anti-PD-1 mAb synergistically increase the secretion of cytotoxic molecules in the supernatant of the co-culture system. (A) The secretion of Granzyme B in the co-culture system of TNBC cells and TNBC cell-specific CTLs treated with control, IL-23, PD-1 mAb, and a combination of IL-23 and PD-1 mAb, respectively. (B) The secretion of IL-2 in the co-culture system of TNBC cells and TNBC cell-specific CTLs treated with control, IL-23, PD-1 mAb, and a combination of IL-23 and PD-1 mAb, respectively. Error bars represent SD, *, *P*<0.05; **, *P*<0.01; ***, *P*<0.001.

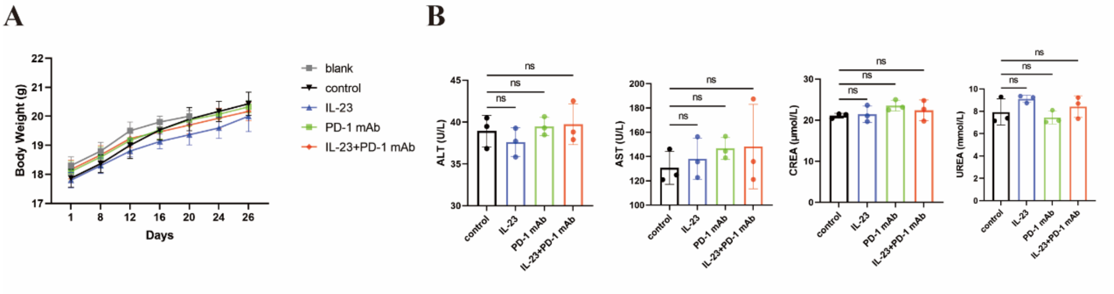

Supplementary Figure 4. The assessment of side effects associated with in vivo treatment using IL-23, PD-1 mAb, and a combination of IL-23 and PD-1 mAb. (A) The quantification of body weight from the indicated groups. (B) The evaluation of liver and kidney functions in mice treated with control, IL-23, PD-1 mAb, and a combination of IL-23 and PD-1 mAb, based on levels of ALT (U/L), AST (U/L), CREA (μmol/L), and UREA (mmol/L).

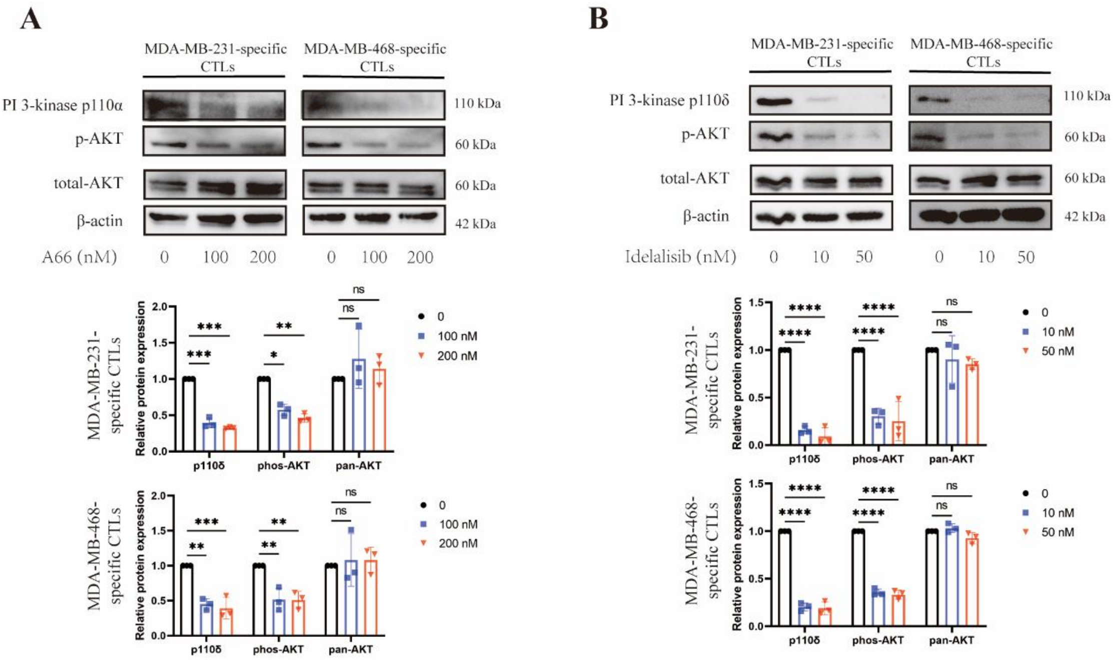

Supplementary Figure 5. The p110α and p110δ contribute to the activation of AKT pathway in TNBC-specific CTLs. (A) The protein expression of PI3-kinase p110α, p-AKT, and total-AKT following the treatment with 0, 100 nM, and 200 nM A66 in TNBC-specific CTLs. Upper panel, the representative images of western blot. Lower panel, quantitative analysis of western blot. The PVDF membranes were cropped at 50 kDa and 70~100 kDa before antibody incubation. The original blots are represented in Supplementary Figure 15. (B) The protein expression of PI3-kinase p110δ, p-AKT, and total-AKT following the treatment with 0, 10 nM, and 50 nM Idelalisib in TNBC-specific CTLs. Upper panel, the representative images of western blot. Lower panel, quantitative analysis of western blot. The PVDF membranes were cropped at 50 kDa and 70~100 kDa before antibody incubation. The original blots are represented in Supplementary Figure 16. Error bars represent SD, *, $P<0.05$; **, $P<0.01$; ***, $P<0.001$; ****, $P<0.0001$.

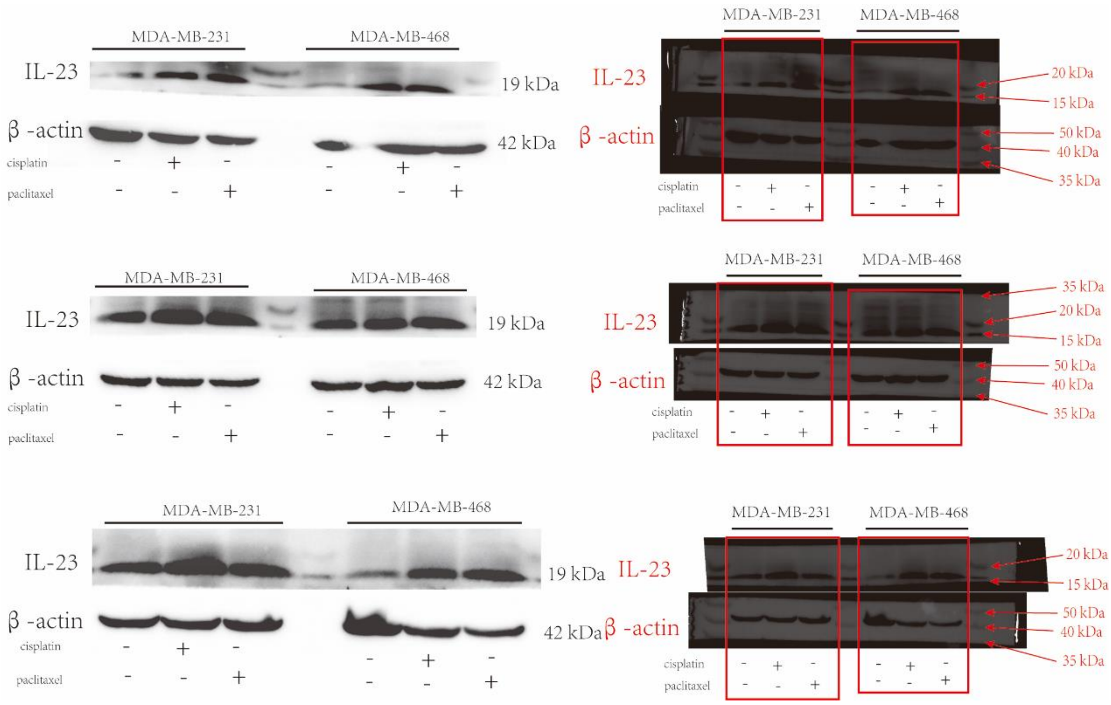

Supplementary Figure 6. The original images of blots exhibited in Figure 1D, as well as the replicates. The blots were cut at 35 kDa prior to hybridization with antibodies.

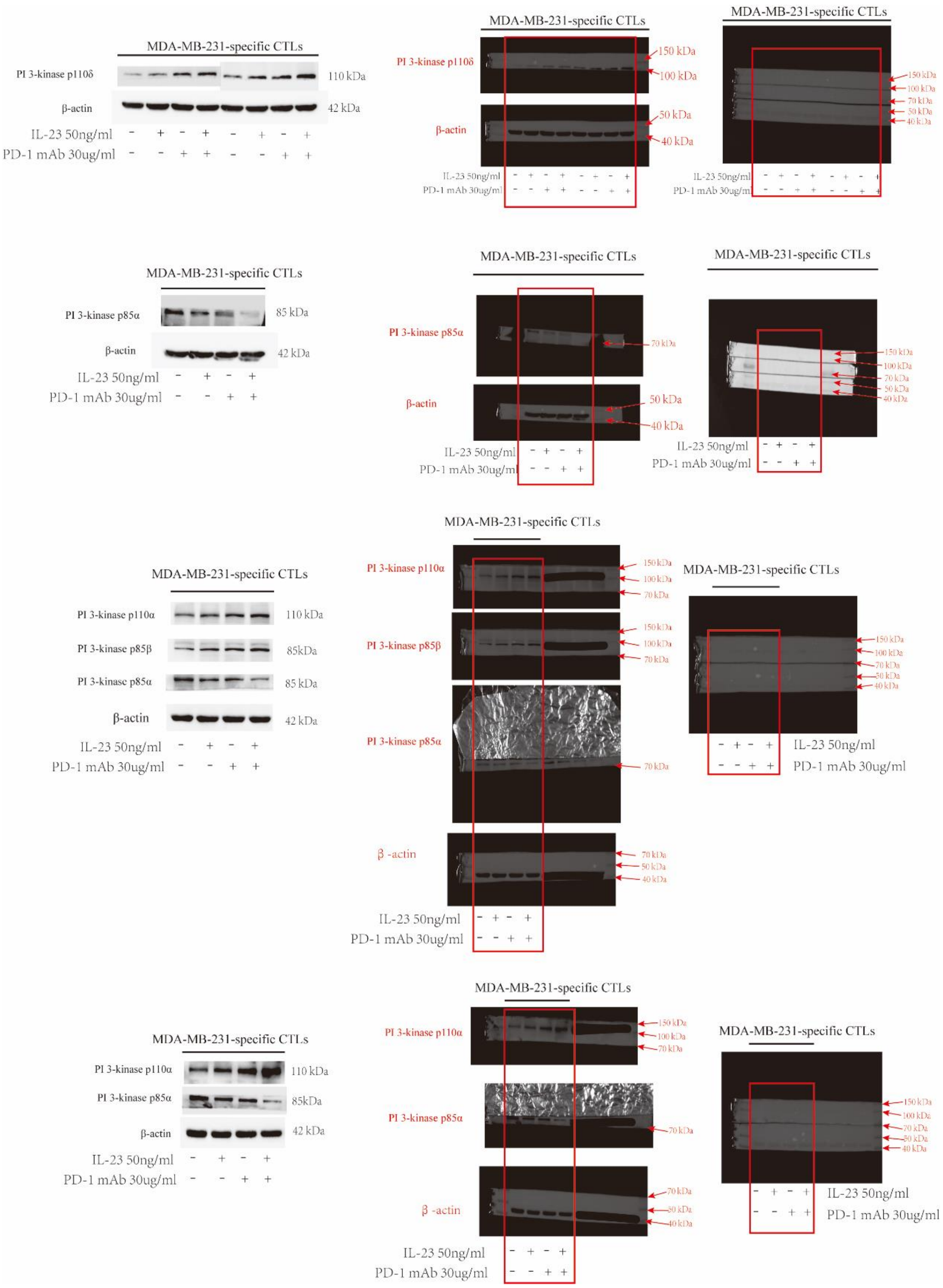

Supplementary Figure 7. The original images of blots exhibited in Figure 4E, 5A, and 5B, as well as the replicates. The blots were cut at 50~70 kDa prior to hybridization with antibodies.

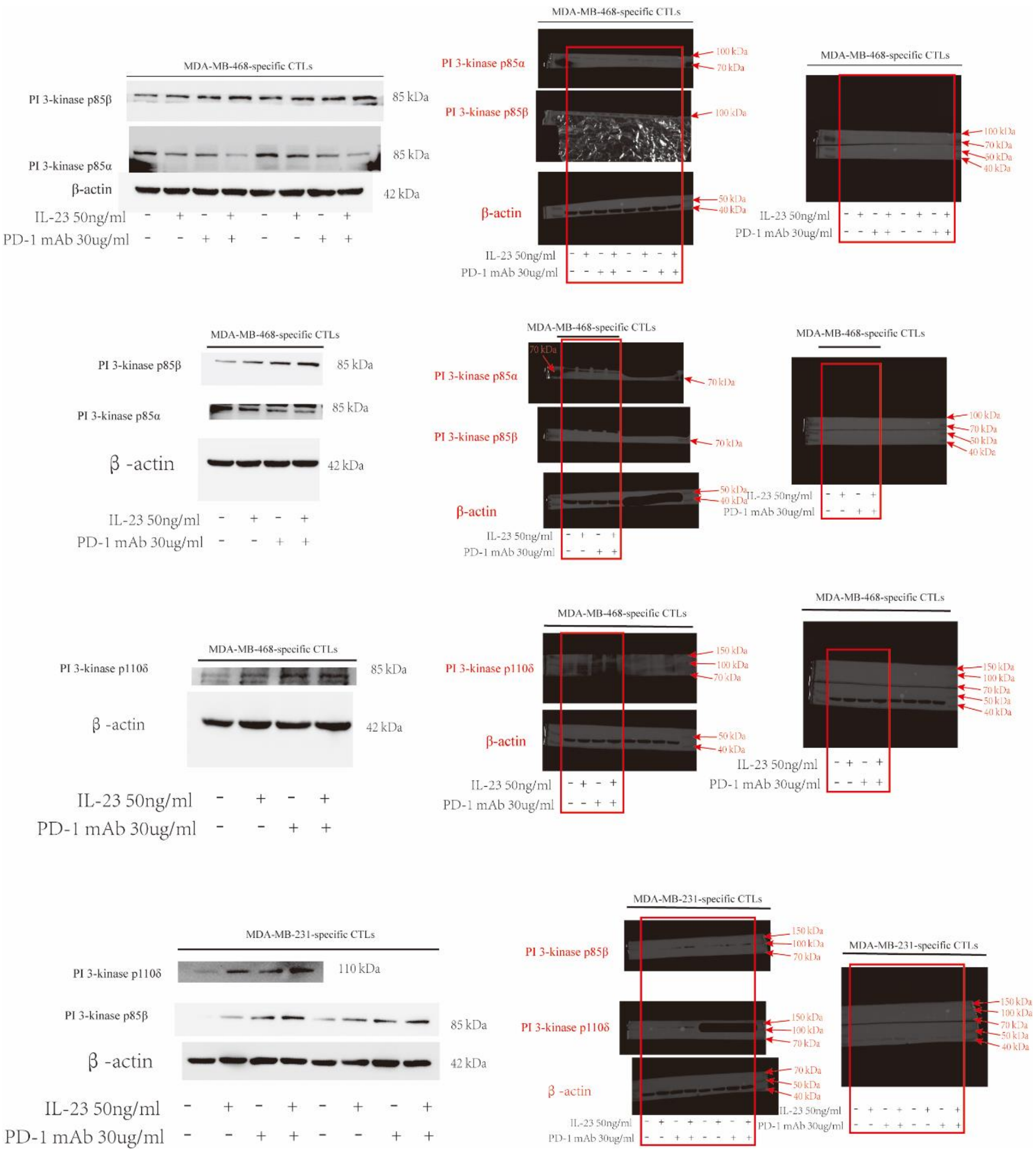

Supplementary Figure 8. The original images of blots exhibited in Figure 4E, 5A, and 5B, as well as the replicates. The blots were cut at 50~70 kDa prior to hybridization with antibodies.

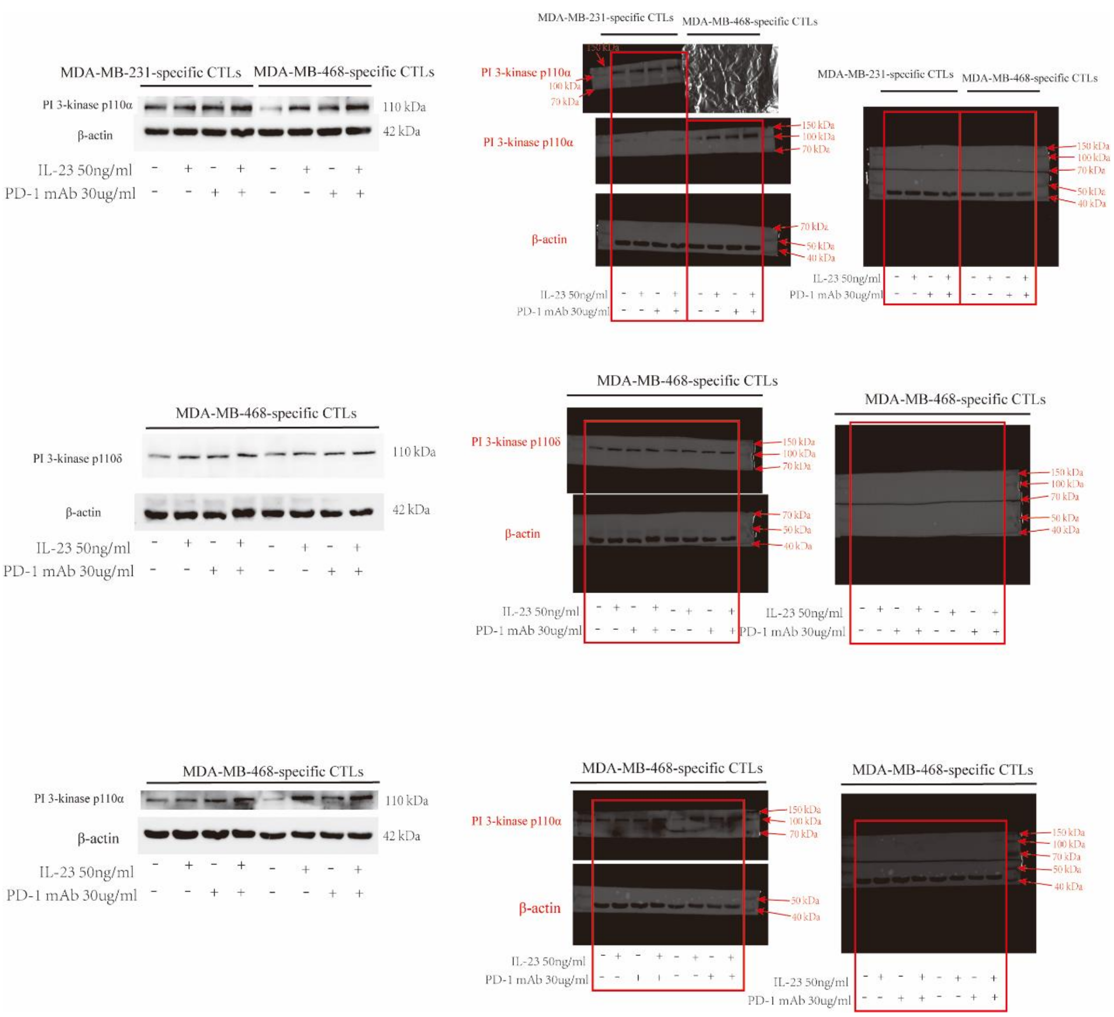

Supplementary Figure 9. The original images of blots exhibited in Figure 4E, 5A, and 5B, as well as the replicates. The blots were cut at 50~70 kDa prior to hybridization with antibodies.

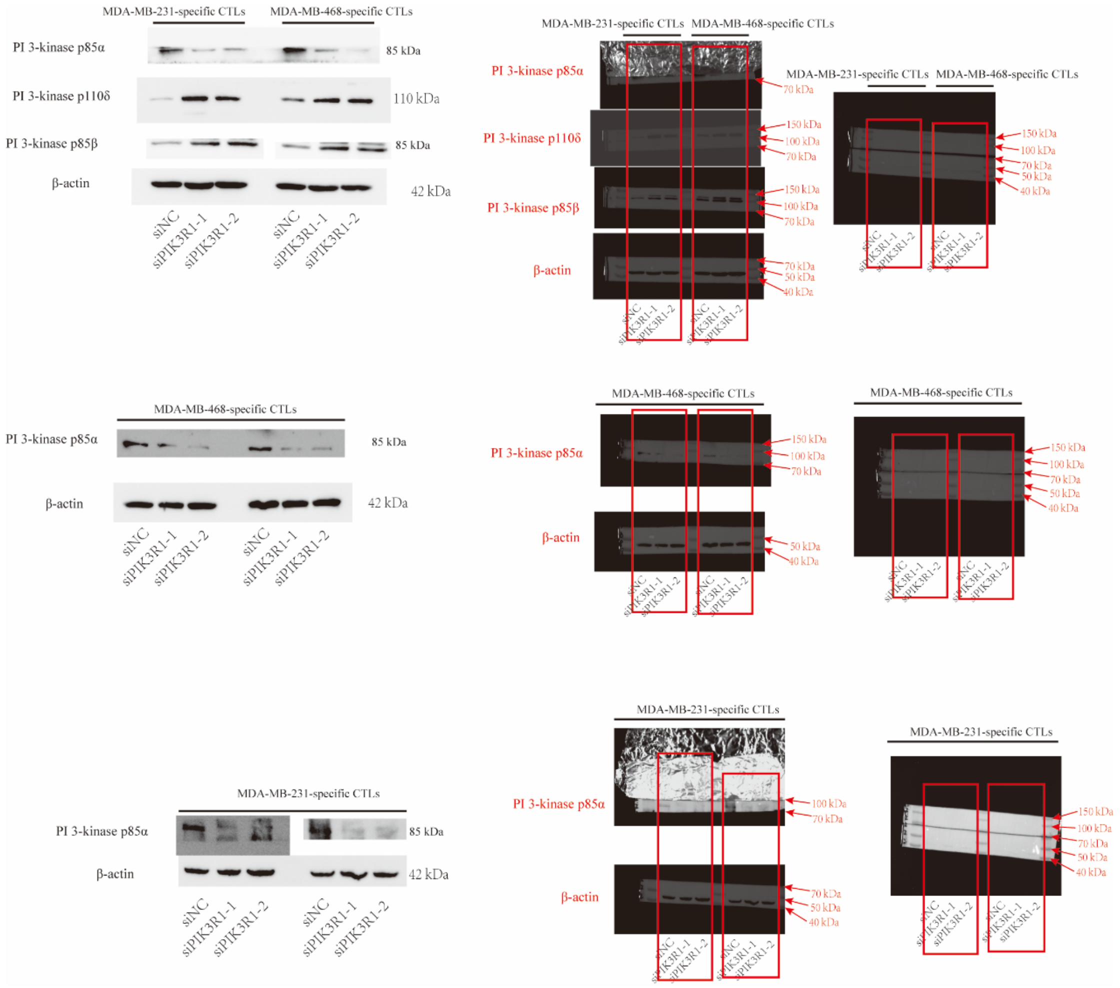

Supplementary Figure 10. The original images of blots exhibited in Figure 5C and 5D, as well as the replicates. The blots were cut at 70 kDa prior to hybridization with antibodies.

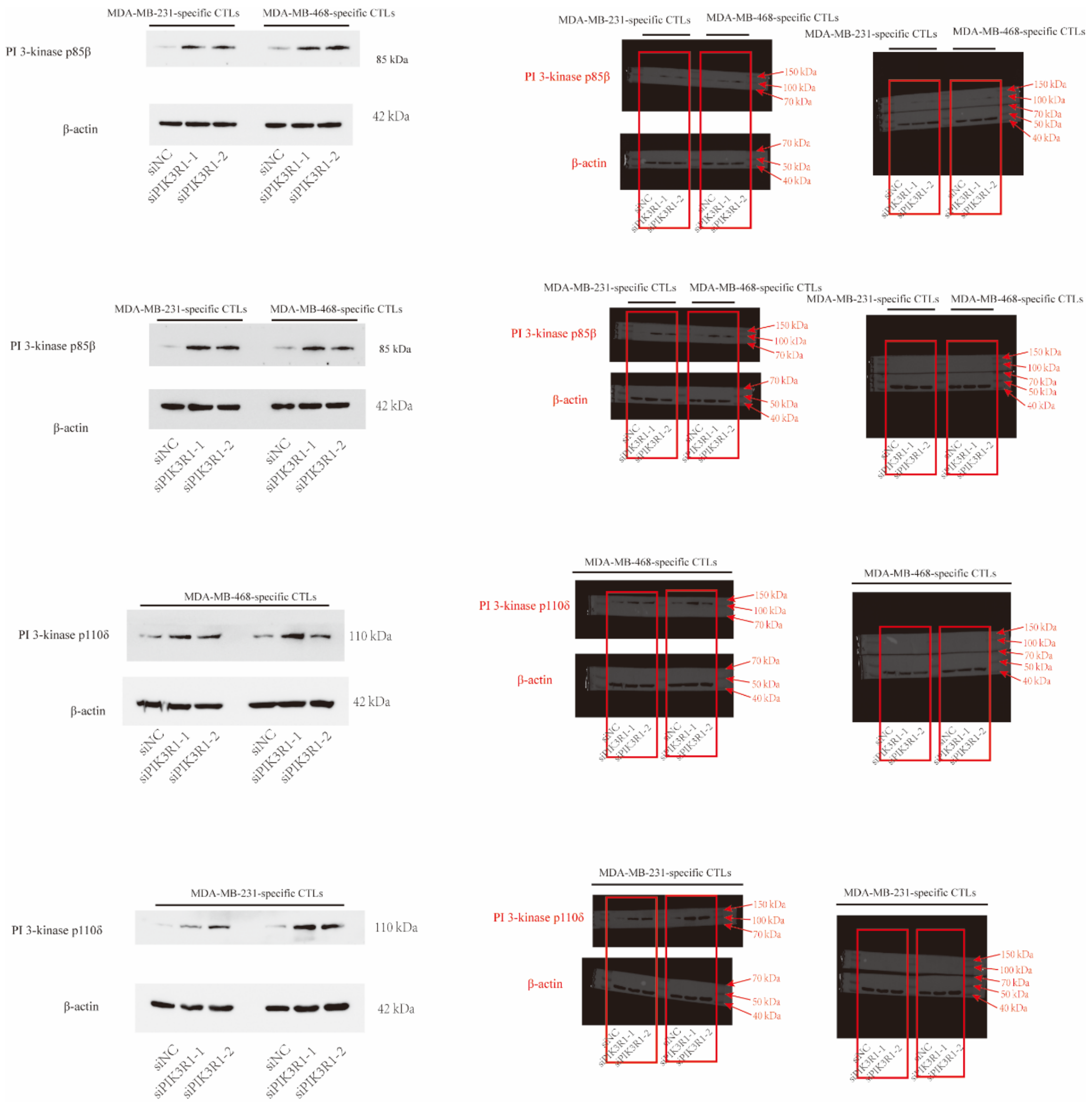

Supplementary Figure 11. The original images of blots exhibited in Figure 5D, as well as the replicates. The blots were cut at 70 kDa prior to hybridization with antibodies.

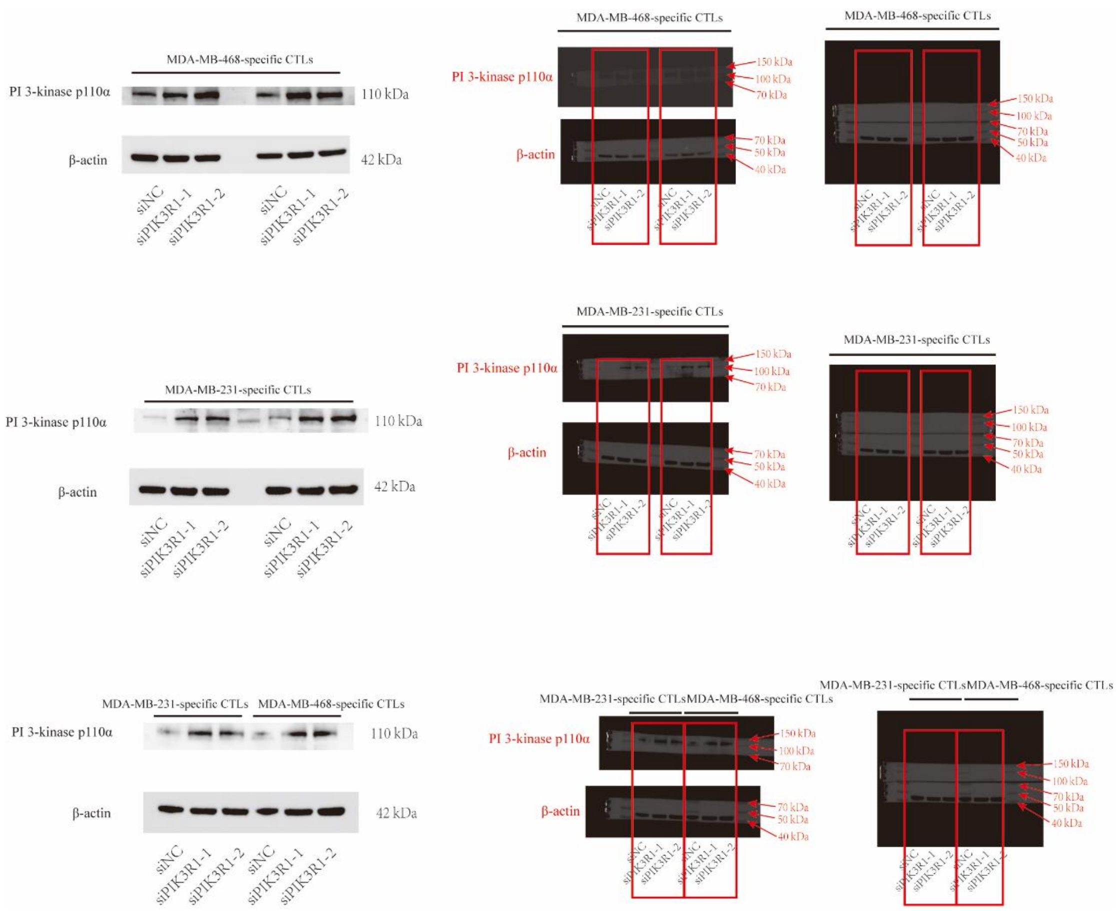

Supplementary Figure 12. The original images of blots exhibited in Figure 5D, as well as the replicates. The blots were cut at 70 kDa prior to hybridization with antibodies.

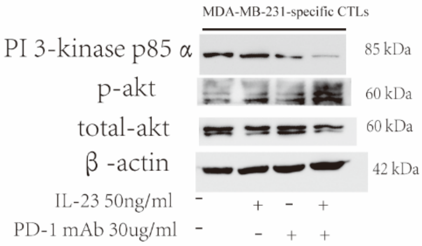
MDA-MB-231-specific CTLs
PI 3-kinase p85 α
p-akt
total-akt
β -actin
85 kDa
60 kDa
60 kDa
42 kDa
IL-23 50ng/ml - + - +
PD-1 mAb 30ug/ml - - + +

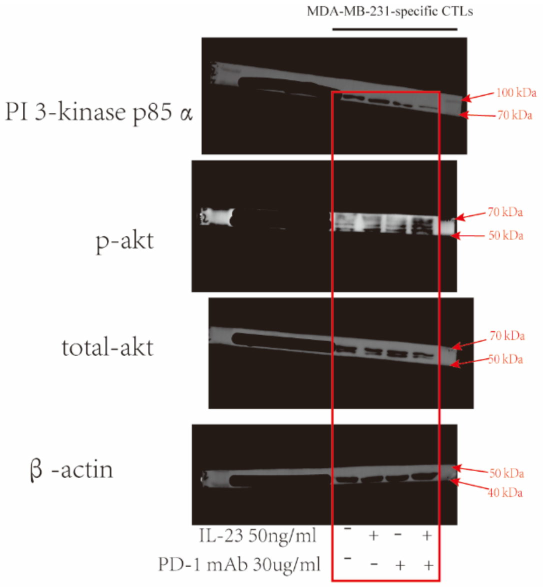
MDA-MB-231-specific CTLs
PI 3-kinase p85 α
p-akt
total-akt
β -actin
100 kDa
70 kDa
70 kDa
50 kDa
70 kDa
50 kDa
50 kDa
40 kDa
IL-23 50ng/ml - + - +
PD-1 mAb 30ug/ml - - + +

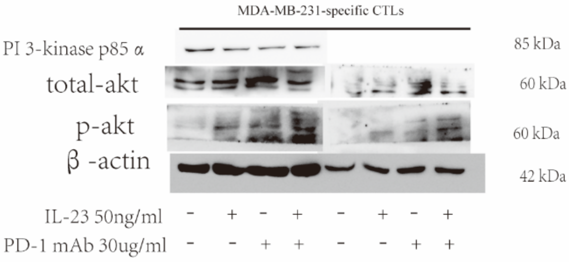
MDA-MB-231-specific CTLs
PI 3-kinase p85 α
total-akt
p-akt
β -actin
85 kDa
60 kDa
60 kDa
42 kDa
IL-23 50ng/ml - + - + - + - +
PD-1 mAb 30ug/ml - - + + - - + +

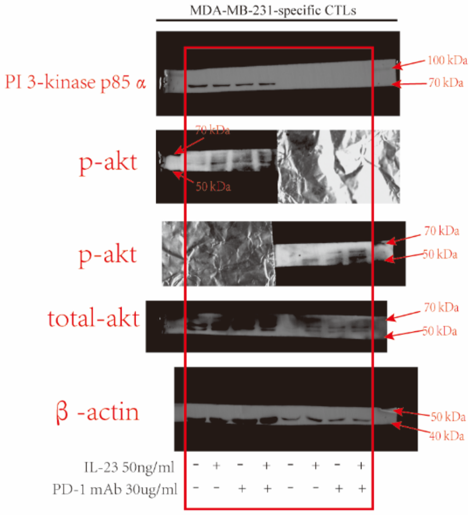
MDA-MB-231-specific CTLs
PI 3-kinase p85 α
p-akt
p-akt
total-akt
β -actin
100 kDa
70 kDa
70 kDa
50 kDa
70 kDa
50 kDa
70 kDa
50 kDa
50 kDa
40 kDa
IL-23 50ng/ml - + - + - + - +
PD-1 mAb 30ug/ml - - + + - - + +

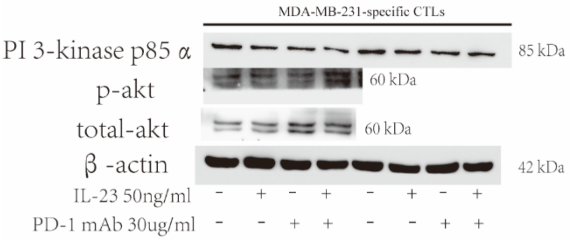
MDA-MB-231-specific CTLs
PI 3-kinase p85 α
p-akt
total-akt
β -actin
85 kDa
60 kDa
60 kDa
42 kDa
IL-23 50ng/ml - + - + - + - +
PD-1 mAb 30ug/ml - - + + - - + +

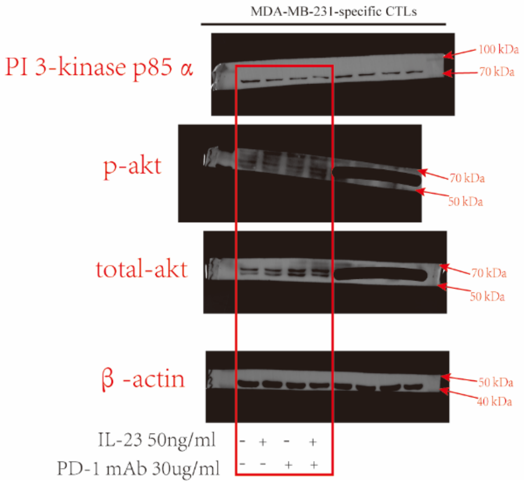
MDA-MB-231-specific CTLs
PI 3-kinase p85 α
p-akt
total-akt
β -actin
100 kDa
70 kDa
70 kDa
50 kDa
70 kDa
50 kDa
50 kDa
40 kDa
IL-23 50ng/ml - + - +
PD-1 mAb 30ug/ml - - + +

Supplementary Figure 13. The original images of blots exhibited in Figure 6A, as well as the replicates. The blots were cut at 50 kDa and 70 kDa prior to hybridization with antibodies.

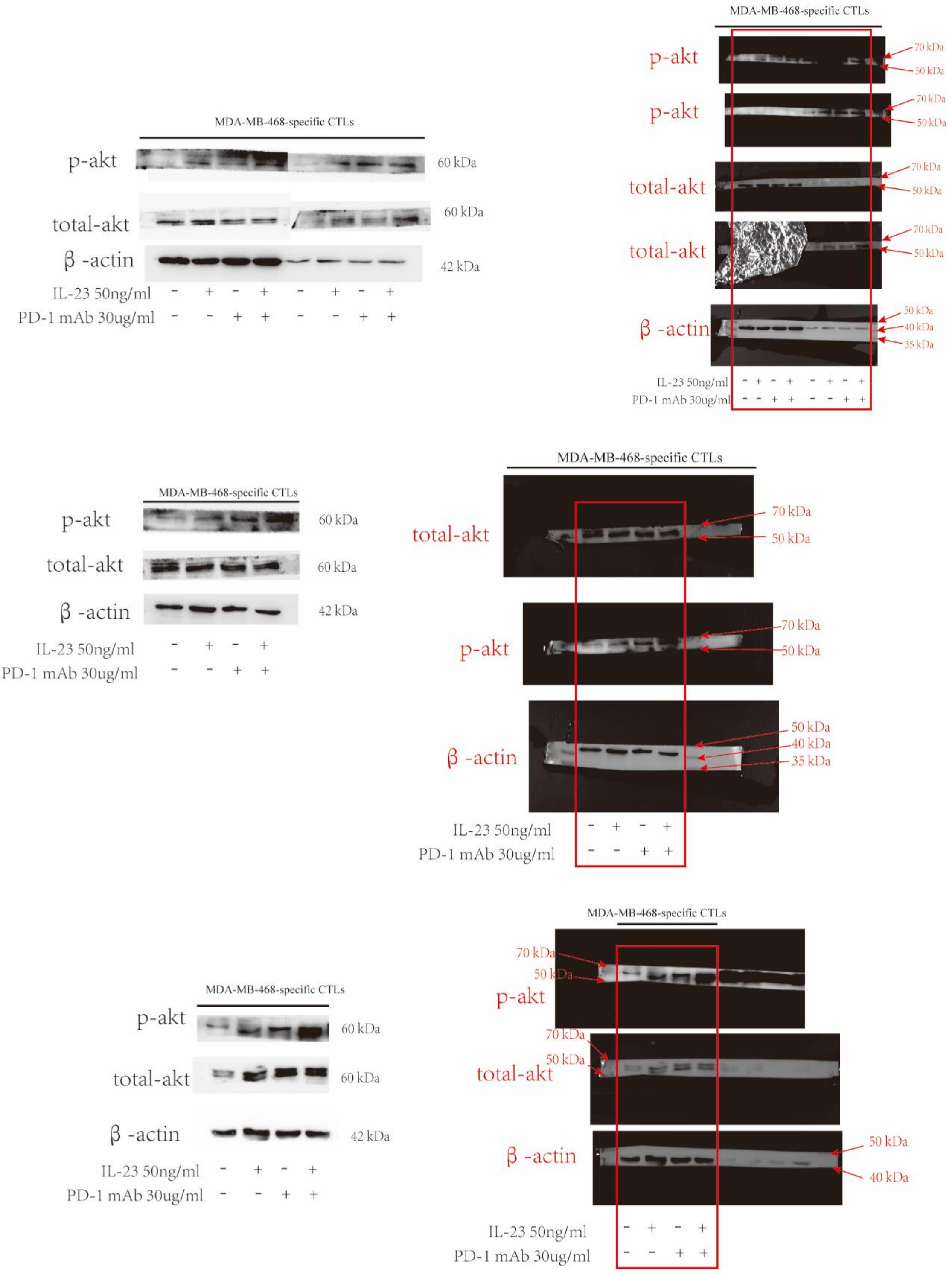

Supplementary Figure 14. The original images of blots exhibited in Figure 6A, as well as the replicates. The blots were cut at 50 kDa and 70 kDa prior to hybridization with antibodies.

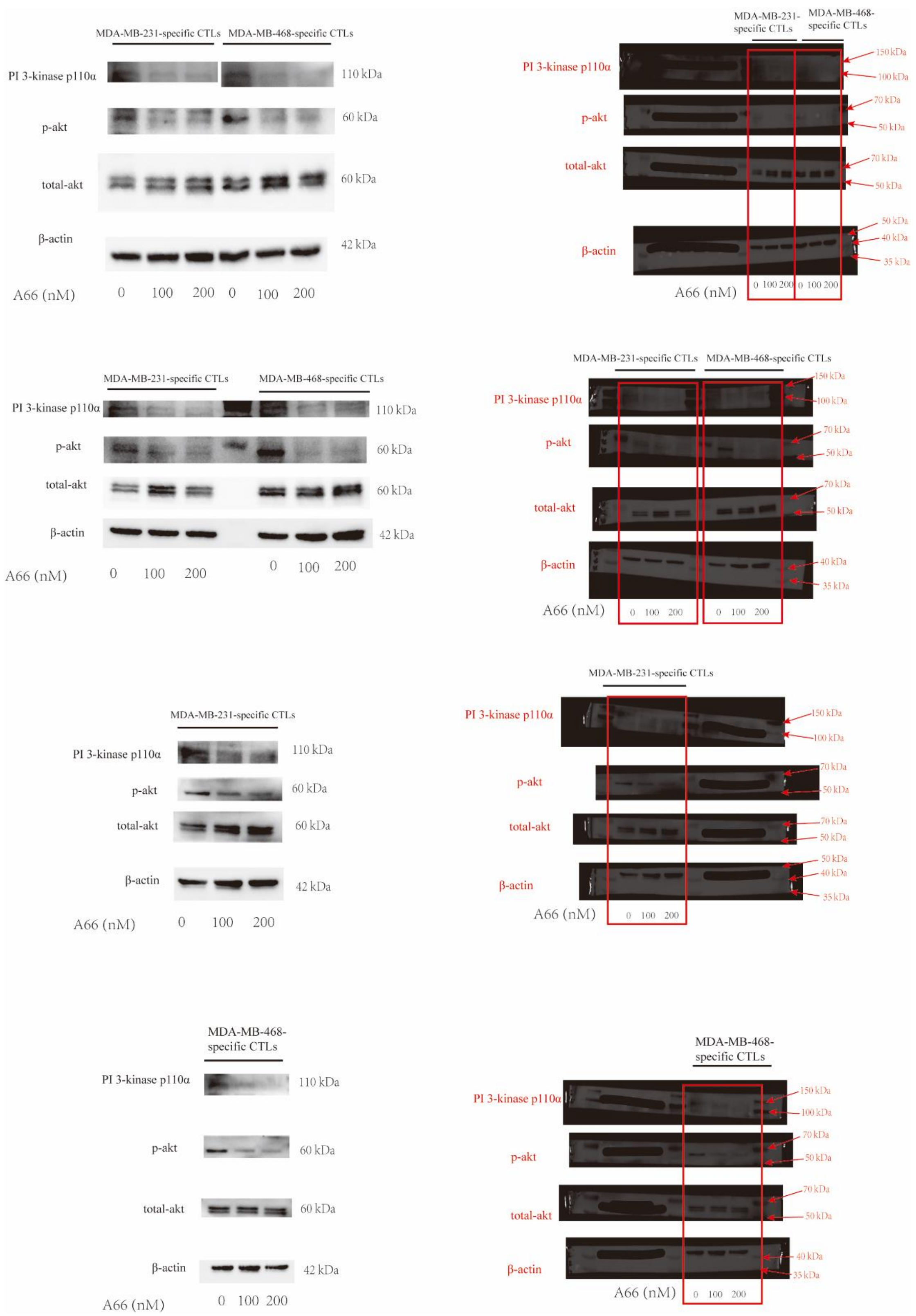

Supplementary Figure 15. The original images of blots exhibited in Supplementary Figure 5A, as well as the replicates. The blots were cut at 50 kDa and 70~100 kDa prior to hybridization with antibodies.

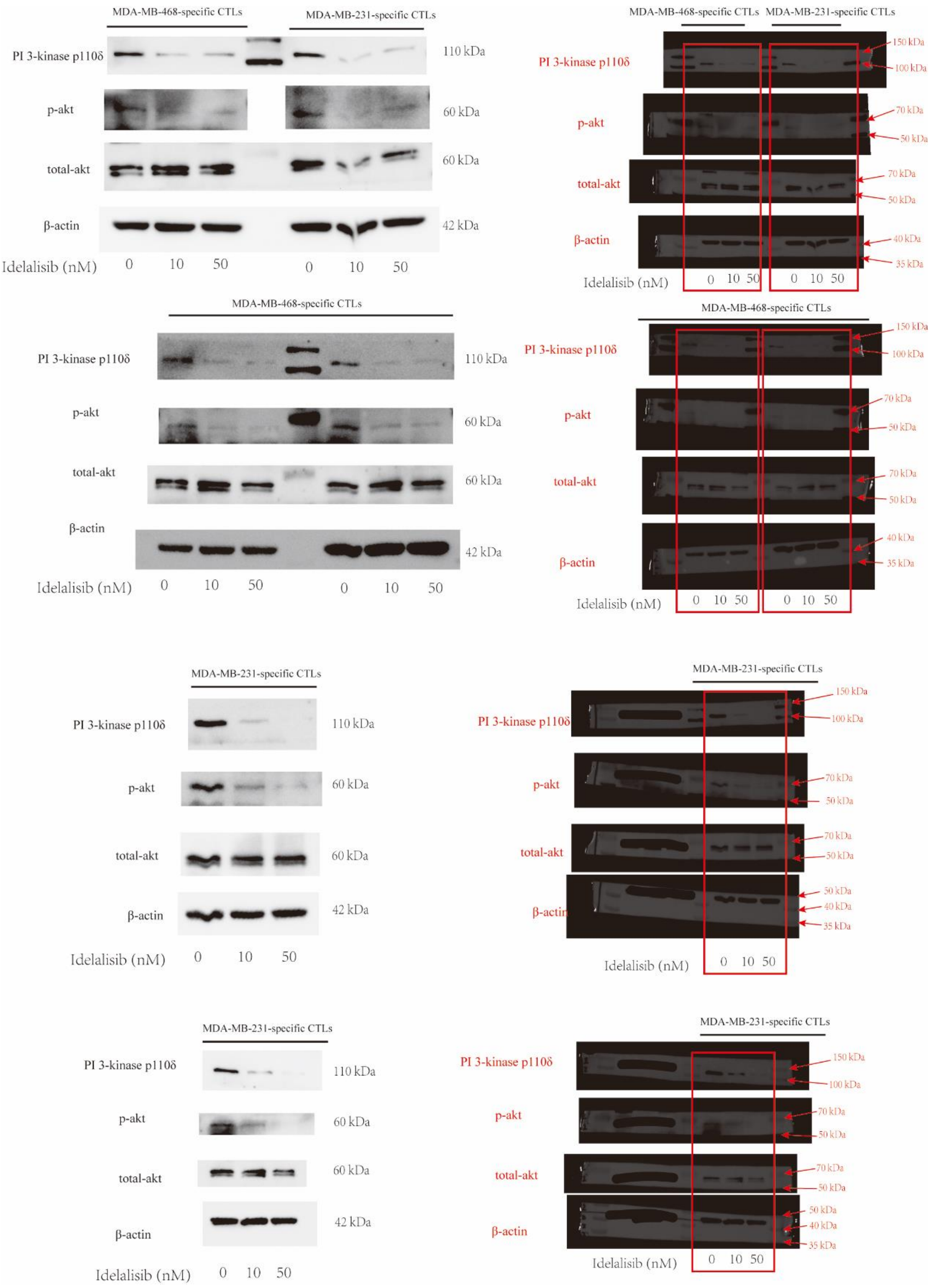

Supplementary Figure 16. The original images of blots exhibited in Supplementary Figure 5B, as well as the replicates. The blots were cut at 50 kDa and 70~100 kDa prior to hybridization with antibodies.

Supplementary tables

Supplementary Table 1. The detailed information of the antibodies and reagents used in this article.

REAGENT	SOURCE	IDENTIFIER
Antibodies		
Mouse anti-IL-23 Antibody (D-12)	Santa Cruz	Cat#sc-271349
Mouse anti-PI 3-kinase p85α Antibody (C-1)	Santa Cruz	Cat#sc-376112
Mouse anti-PI 3-kinase p85β Antibody (H-1)	Santa Cruz	Cat#sc-515646
Mouse anti-PI 3-kinase p110α Antibody	Proteintech	Cat#67071-1-Ig
Mouse anti-PI 3-kinase p110β Antibody (C-8)	Santa Cruz	Cat#sc-376641
Mouse anti-PI 3-kinase p110δ Antibody (A-8)	Santa Cruz	Cat#sc-55589
Rabbit anti-Phospho-Akt (Ser473) Antibody	Cell Signaling Technology	Cat#9271S
Mouse anti-AKT Monoclonal antibody	Proteintech	Cat#60203-2-Ig
Mouse anti-Beta Actin Monoclonal antibody	Proteintech	Cat#66009-1-Ig
Mouse anti-IL-23a Monoclonal antibody	Proteintech	Cat#66196-1-Ig
Rabbit anti-human CD8 alpha mAb	Abcam	Cat#ab245118
Mouse anti-pan Cytokeratin mAb	Abcam	Cat#ab7753
Mouse anti-Granzybe B mAb (GB7)	Santa Cruz	Cat#sc-56119
Mouse anti-Ki67 mAb	Santa Cruz	Cat#sc-23900
Critical commercial reagent		
Percoll	Sigma-Aldrich	Cat#P4937
CD8 MicroBeads, human	Miltenyi Biotec	Cat#130-045-201
Recombinant Human GM-CSF	PEPROTECH	Cat#300-03
Recombinant Human IL-4	PEPROTECH	Cat#200-04
Recombinant Human IL-2	PEPROTECH	Cat#200-02
Recombinant Human IL-23	PEPROTECH	Cat#200-23
Pembrolizumab	MSD	
CD3 Monoclonal Antibody (OKT3)	Thermo Fisher	Cat#16-0037-81
CD28 Monoclonal Antibody (37.51)	Thermo Fisher	Cat#MA1-10172
Human Granzyme B Quantikine ELISA Kit	R&D Systems	Cat#DGZB00
Human IL-2 Quantikine ELISA Kit	R&D Systems	Cat#QK202
CD3 FITC/CD8 PE/CD45 PerCP/CD4 APC reagent	BD Biosciences	Cat#340499
AKT inhibitor VIII (AKTi-1/2)	MedChemExpress	Cat#HY-10355
TRIzol	Thermo Fisher	Cat#15596026
PrimeScript RT reagent Kit	Takara	Cat#RR037
SYBRTM Green PCR	Thermo Fisher	Cat#4309155
Protein marker	Epizyme Biotech	Cat#WJ101
